# Polymer physics indicates chromatin folding variability across single-cells results from state degeneracy in phase separation

**DOI:** 10.1038/s41467-020-17141-4

**Published:** 2020-07-03

**Authors:** Mattia Conte, Luca Fiorillo, Simona Bianco, Andrea M. Chiariello, Andrea Esposito, Mario Nicodemi

**Affiliations:** 1 Dipartimento di Fisica, Università di Napoli Federico II, and INFN Napoli, Complesso Universitario di Monte Sant’Angelo, 80126 Naples, Italy; 2 Berlin Institute for Medical Systems Biology, Max-Delbrück Centre (MDC) for Molecular Medicine, Berlin, Germany; 3 Berlin Institute of Health (BIH), MDC-Berlin, Berlin, Germany

**Keywords:** Epigenetics, Chromatin structure, Biological physics

## Abstract

The spatial organization of chromosomes has key functional roles, yet how chromosomes fold remains poorly understood at the single-molecule level. Here, we employ models of polymer physics to investigate DNA loci in human HCT116 and IMR90 wild-type and cohesin depleted cells. Model predictions on single-molecule structures are validated against single-cell imaging data, providing evidence that chromosomal architecture is controlled by a thermodynamics mechanism of polymer phase separation whereby chromatin self-assembles in segregated globules by combinatorial interactions of chromatin factors that include CTCF and cohesin. The thermodynamics degeneracy of single-molecule conformations results in broad structural and temporal variability of TAD-like contact patterns. Globules establish stable environments where specific contacts are highly favored over stochastic encounters. Cohesin depletion reverses phase separation into randomly folded states, erasing average interaction patterns. Overall, globule phase separation appears to be a robust yet reversible mechanism of chromatin organization where stochasticity and specificity coexist.

## Abstract (web summary)

The molecular and physical mechanisms underlying chromatin folding at the single DNA molecule level remain poorly understood. Here, the authors use polymer modeling to investigate the conformations of two 2Mb-wide DNA loci in normal and cohesin depleted cells, and provide evidence that the architecture of the studied loci is controlled by a thermodynamics mechanism of polymer phase separation whereby chromatin self-assembles in segregated globules.

## Introduction

In the cell nucleus, chromosomes are folded into a complex 3-dimensional (3D) architecture^1–5^ including a hierarchy of interactions, from loops^6^ and TADs^7,8^ to, above the megabase scale, metaTADs^9^ and A/B compartments^10^ as revealed by population-averaged contact maps^6,10–12^. Such an organization serves important functional purposes as genes and enhancers have to form specific physical contacts to regulate transcription. TADs, for instance, are thought to act as insulating structures, spatially confining the activity of enhancers to their proper targets^2,3,5^.

Different molecular factors and mechanisms have been involved in the 3D organization of chromatin. CTCF binding sites and cohesin have been proposed to shape loops and TADs^6^, for example via the cohesin/CTCF based loop-extrusion model^13–15^. However, while acute depletion of CTCF or cohesin leads to loop loss in bulk Hi-C data, signals persist at the compartment level and finer contact patterns remain within former loops or TADs^16–18^. Compartments A and B are known to correlate to different transcriptional states^10^, and homotypic interactions between active and poised gene promoters, linked respectively to Pol-II-S2p and PRC2, have been observed at the Mb scale and traced back to phase separation mechanisms^19–21^. Indeed, phase separation has emerged as a paradigm of cell organization^22^ and of transcriptional control^23^, as combinations of Pol-II with transcription factors and coactivators, such as Mediator, appear to form condensates^24–26^, or more fleeting interactions^27^, linked to gene regulation^23,28–30^. Yet, it remains unclear how those mechanisms act and combine to shape chromatin architecture.

Single-cell Hi-C experiments, for example, have highlighted the stochastic nature of TADs and the strong variability of their contacts^31–34^. Recent super-resolution imaging approaches have shown that TAD-like structures are present in single cells with chromatin folded in globular 3D conformations, but they broadly vary from cell to cell^35–39^. In particular, TAD boundaries were discovered to occur with nonzero probability at all genomic positions and to have enrichments associated to only a subset of the CTCF sites in the considered regions^37^. In addition, cohesin depletion was found to leave contact patterns at the TAD-scale intact in single cells, albeit domain boundaries become equally likely to locate at any genomic position, hence abolishing TADs at the population-average level. That hinted that chromatin contacts could arise from mechanisms distinct from the loop-extrusion^37^.

**Fig. 1:**
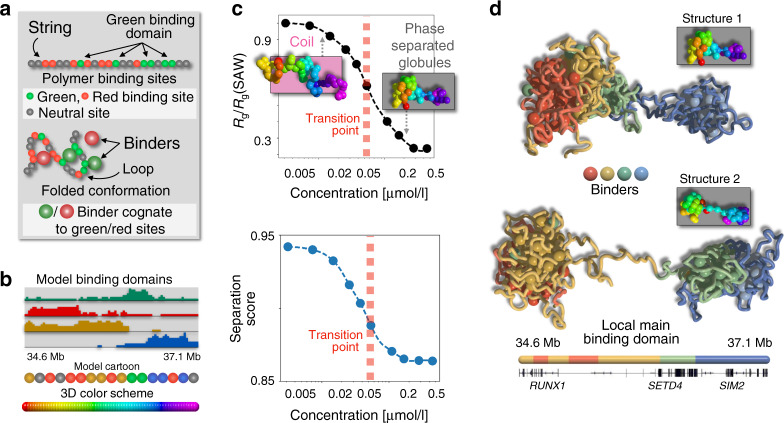
The model phase transition from a coil to a globule phase separated state. **a** Cartoon of the Strings and Binders (SBS) polymer model of chromatin showing the specific binding sites along the chain (top) and a 3D conformation of the system folded by the action of cognate binders (bottom). **b** The SBS model of the studied chr21:34.6-37.1 Mb locus in human HCT116 cells has four types of binding sites, forming four binding domains each represented with a different color. Their genomic location and abundance are shown. A cartoon is sketched (bottom) of the model polymer chain and the color scheme used in the 3D representations in **c**). **c** Upon increasing the binder concentration, the model has a phase transition from a coil to a globule phase separated state, a more condensed structure made of partially separated globules, as signaled by a sharp decrease of the system order parameters, respectively the equilibrium gyration radius *R*_g_ (top) and average separation score (bottom). **d** The intrinsic degeneracy of the globule phase separated state, enhanced by the overlapping genomic organization of the binding domains, corresponds to a variety of 3D conformations: two structures are shown where, for example, the green binding domain collapses respectively onto the brown and the blue domain.

Those diverse results raise questions on the nature and origin of contact patterns in single DNA molecules. Are there other folding mechanisms beyond loop-extrusion? How does phase separation act? If interactions are stochastic, how is specificity controlled? What is the origin of structural variability across cells and in time? To attack those questions, here we use a chromatin model from polymer physics to derive predictions about DNA single-molecule 3D structures that we compare with super-resolution imaging data in single cells^37^. In particular, we investigate two 2 Mb wide DNA regions in human HCT116 and IMR90 cells, where bulk Hi-C^6,16^ and single-cell imaging^37^ data are available. To reconstruct chromatin 3D conformations different computational methods^40–43^ and polymer models have been developed^13–15,19,20,44–52^. In this work, we focus on the textbook scenario where contacts between distal DNA binding sites are established by diffusing cognate binding factors, as described by the Strings and Binders (SBS) polymer physics model of chromatin^19,20,47^ (Fig. 1a). By machine learning from only Hi-C data^6,16^, we infer the genomic location of the putative binding sites of the SBS polymer model of the loci of interest, which are shown to correlate with specific combinations of known chromatin organizing factors. Next, by Molecular Dynamics (MD) simulations we derive a thermodynamics ensemble of single-molecule 3D structures of those loci.

As dictated by polymer physics^53^, we find that the model 3D conformations fall in two main folding classes corresponding to its thermodynamics phases, the coil, i.e., randomly folded, and the globule state, where distinct globules self-assemble along the chain by the interactions of cognate binding sites. According to the concentration or affinity of binders, the system switches from one to the other state via a phase transition mechanism of polymer phase separation. We show that those 3D structures recapitulate bulk Hi-C data and we validate model predictions on single-molecule 3D conformations against independent imaging single-cell data in both wild-type (WT) and cohesin depleted cells^37^. The consistent agreement provides evidence that, in the studied loci, chromatin folding is explained at the single-molecule level by such a thermodynamics mechanism, different from loop-extrusion. In particular, in the model of WT cells we find that the loci fold mostly in globule conformations, whose inherent thermodynamics degeneracy manifests in the broad variability of TAD-like domains across single-molecules. We also explore the time dynamics of chromatin structure at the single molecule level. Globule formation produces dynamic, yet stable local compact environments highly favoring close contacts between sites enriched for cognate binding sites, within and, less frequently, across globules. That exemplifies how stochasticity of DNA interactions can coexist with contact specificity. Acute cohesin depletion reverses phase separation into the coil state in the majority of cells, producing much more variable and transient contact patterns.

## Results

### Model phase transition to the globule phase separated state

We focused, first, on modeling a 2.5 Mb DNA region (chr21:34.6–37.1 Mb) in human HCT116 cells. The SBS is a simplified, coarse-grained model where a chromatin filament is represented as a self-avoiding chain of beads and along the chain are located specific binding sites for cognate, diffusing molecular binders^19,20,47^ (Fig. 1a), as well as unspecific binding sites. To check that our general conclusions are robust, as expected from Statistical Mechanics^53^, in our study we explored a spectrum of specific and unspecific affinities between binders and binding sites in the weak biochemical energy range, respectively from 3.1 to 8.0*K*_B_*T* (for simplicity equal across the different types) and from 0 to 2.7*K*_B_*T* (“Methods”).

To infer the genomic location and the types of the putative binding sites of the SBS polymer model of the locus, we developed a machine learning procedure (“Methods” and Supplementary Fig. 1) based on the PRISMR approach^50^, which employs as input only bulk Hi-C data^16^, with no use of epigenetic tracks to avoid biases toward a subset of factors. The procedure returns four distinct types of specific binding sites (visually represented by different colors, Fig. 1b), each defining a binding domain. After setting the affinities, the system is investigated at different binder concentrations (equal for all types), from 0 to 0.5 μmol/l, by MD simulations to derive, for each different concentration, a thermodynamic ensemble of single-molecule 3D conformations of the model of the locus.

**Fig. 2:**
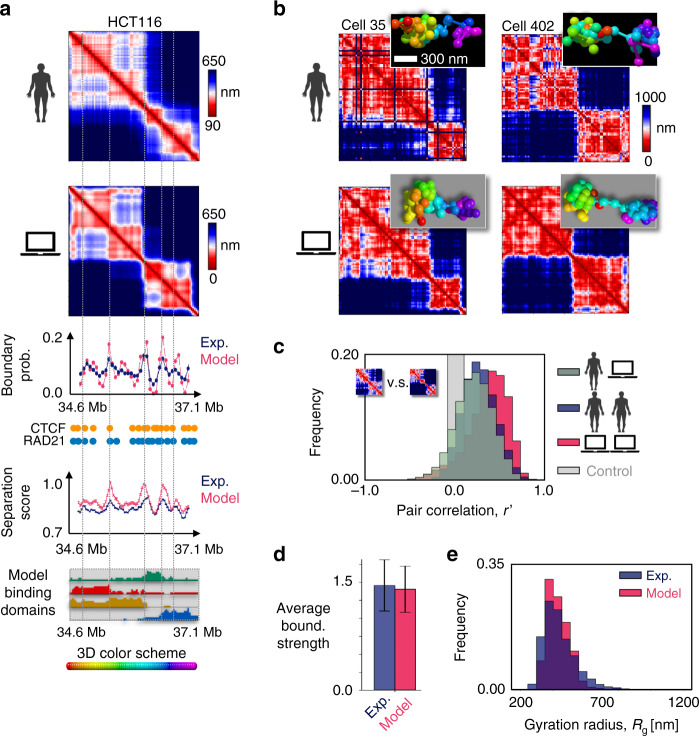
Phase separation degeneracy explains variability of single-molecule conformations. **a** In the considered 2.5 Mb wide locus chr21:34.6Mb-37.1 Mb of human HCT116 cells, the model median distance matrix in the globule separated state compares well against imaging data^37^ (top, the Pearson and genomic-distance corrected correlations are, respectively, *r* = 0.95 and *r*′ = 0.84). The probability of a domain boundary in 3D conformations along the locus (middle, model-experiment correlation *r* = 0.79) and the corresponding separation score (model-experiment *r* = 0.85) also match. The location of ChIP-seq CTCF (orange circles) and cohesin (RAD21, blue circles) sites^58^, and the intensity of the model four types of specific binding sites along the locus (bottom, as in Fig. 1b) are also shown. The vertical dotted lines are drawn to help comparing the panels. **b** Consistently, an all-against-all comparison of single-cell imaged^37^ (top) and model predicted (bottom) 3D structures by the RMSD method shows that all imaged conformations statistically map onto model single molecules in the globule state (bottom, see text and Supplementary Figs. 5b, 6). The varying TAD-like domains result from the intrinsic conformational degeneracy of such a thermodynamic state. **c** The degree of variability of single-molecules is measured by the genomic-distance corrected correlation, *r*′, of pairs of distance matrices. The distribution of *r*′ between pairs of imaged structures (blue, average *r*′ = 0.27) is statistically not distinguishable from the *r*′ distribution between imaged and model distance matrices (dark gray, two-sided Mann–Whitney *p* value = 0.19). The model-model *r*′ distribution is in red and in light gray a control. **d** The model and experimental average boundary strength (error bars s.d.), and **e** the gyration radius distributions are also not distinguishable (two-sided Mann–Whitney *p* value = 0.40). Overall, the polymer globule phase separated state of the model returns single molecule structures with features consistent with both single-cell imaging and bulk Hi-C (Supplementary Fig. 4a) data.

Upon increasing the binder concentration, we find that at a characteristic threshold (Fig. 1c) the polymer undergoes a thermodynamics phase transition from a coil to a globule phase separated state^53^, corresponding to a sharp conformational rearrangement. In our HCT116 main case study, the threshold concentration is about 50 nmol/l (Fig. 1c) and, more generally, for the explored weak biochemical affinities it falls in the fractions of μmol/l range^47^, values compatible with transcription factor concentrations. As known in block-copolymers^20,54,55^, in the coil state entropic forces keep the polymer in randomly folded conformations, while in the phase separated state attractive forces thermodynamically prevail and the different binding domains self-assemble by action of (and along with) their cognate binders in more compact and partially separated globules, as signaled respectively by a sharp drop in the gyration radius, *R*_g_, and separation score, the order parameters of the system (as well as in its binding energy, Supplementary Fig. 2). Differently from usual linear block-copolymers, though, the separation of the globules is only partial because of the overlapping genomic distribution of the underlying binding sites that increases the degeneracy of the system microstates, which can fold in a multiplicity of 3D conformations (Fig. 1d). The self assembly of globules is guided by the nontrivial genomic arrangement of the four binding domains of the model that are enriched each in a distinct, successive genomic region and hence form the polymer core globules, which result into the main TAD-like structures of the median distance map of the model (Fig. 2a).

To gain insights into the molecular nature of the inferred model binding sites, which are responsible of folding, we correlated their genomic positions with available epigenetic data in the same cell type^16^ (Supplementary Fig. 3). Interestingly, we find that each single binding type (color) has statistically significant Pearson correlations with a specific combination of known architecture organizing factors. The first putative binding domain (green, in Fig. 1b) correlates mainly with the CTCF/Smc1 (Cohesin) system, the second one (red) with active marks (e.g., H3K27ac and transcription factors) and less with Smc1, the third (brown) with repressive marks (e.g., H3K27me3), whereas the fourth (blue) with H4K16ac and specific transcription factors.

Summarizing, our polymer model undergoes a phase transition from a coil to a phase separated globular state as the number of binders (or affinity strength) grows above a threshold point. For a given binder concentration, the system can fold in a variety of 3D conformations, not just in a unique, naïve structure. As dictated by polymer physics^53^, however, the system 3D conformations fall in two main folding classes corresponding to its thermodynamics phases, the coil and the globule separated states. Folding is controlled by the system binding sites and cognate binders, each type correlated with a different combination of chromatin architecture factors.

### Model validation against independent imaging distance data

To check that the model derived 3D structures recapitulate the Hi-C data used to infer its putative binding sites, we computed the average contact matrix in the two thermodynamic phases. While in the coil state the contact matrix is structureless, in the globular state it exhibits a pattern of TADs and sub-TADs similar those in Hi-C data (Supplementary Fig. 4a), as highlighted by the high Pearson, *r* = 0.88, and genomic distance corrected Pearson correlation coefficient, *r*′ = 0.68, between model and Hi-C contact data.

In a first validation of our model and of its Hi-C inferred putative binding sites, we also compared its predictions about the locus median distance matrix in the globular state against independent super-resolution imaging data^37^ (Fig. 2a) and found that they have a Pearson, *r* = 0.95, and distance-corrected correlation, *r*′ = 0.84, even higher than correlations with Hi-C data. Hence, the basic physics ingredients of our polymer model and its inferred binding sites are sufficient to recapitulate bulk Hi-C and independent imaging data.

Next, to demonstrate that our model provides a bona fide representation of chromatin conformations in single cells, we performed an all-against-all comparison between its predicted single-molecule 3D structures and single-cell 3D structures from imaging data^37^ (Fig. 2b). By use of a method^33^ that finds the optimal rotation between two centered 3D structures to minimize the mean squared deviation (RMSD) of their coordinates (Supplementary Fig. 5a), each experimental 3D structure was univocally associated to a corresponding model 3D structure by searching for the least RMSD (Supplementary Fig. 6a). Consistent with the results on average contact and distance matrices, in the HCT116 case we find that all experimental structures map onto model conformations in the thermodynamics globule state (Supplementary Fig. 5b). To test the significance of the association, we compared the RMSD distribution of the experiment-model optimal matches to the RMSD distribution of pairwise comparisons between experimental structures (null model): the two distributions are statistically different (Mann–Whitney test *p* value = 0) with only 2% of entries of the former falling above the first quartile of the latter (Supplementary Fig. 6b). In addition, we find that each model globule conformation is significantly associated to at least one experimental structure, showing that the model well represents the experimental ensemble.

### Degeneracy in phase separation explains variability of single-molecule conformations

To further validate our model, we compared the architectural features of its predicted single-molecule 3D conformations against single-cell 3D structures from imaging^37^ (Fig. 2b). In single cell experiments, the locus folds in spatially segregated globules, as highlighted by the separation score as a function of the genomic coordinate (Fig. 2a), which produce the TAD and sub-TAD-like domains of the distance matrix. However, the 3D structures are broadly varying across single cells, and TAD boundaries are found to be spread along the entire locus (see the boundary probability in Fig. 2a). We aimed to test whether the model ensemble of single-molecule conformations has features similar to those found in single-cell experiments and whether it has a similar variability (Fig. 2b).

First, we found that: (i) the model derived TAD-like boundary probability and, (ii), separation score along the locus are very similar to the experimental ones (respectively *r* = 0.79 and *r* = 0.85, Fig. 2a); (iii), the average boundary strengths are similar (Fig. 2d); (iv) the average boundary probabilities and, (v), the boundary strength distributions are similar too (Supplementary Fig. 7a, b), albeit there are no free parameters in all those comparisons. In addition, the gyration radius distributions of the model and experiment are also found to be statistically not distinguishable from each other (Mann–Whitney *p* value = 0.40, Fig. 2e). Conversely, a control block-copolymer model with four non-intertwining binding domains designed specifically to reproduce the main TAD-like structures visible in bulk Hi-C data, which has also a coil-to-globule transition, was found to poorly reflect the complexity of the observed contact patterns (Supplementary Fig. 8).

Second, to quantify the variability of experimental single-cell 3D structures, we measured the distance-corrected correlation, *r*′, between pairs of single-cell distance matrices, and found that it has a broad distribution with an average correlation *r*′ = 0.27 (Fig. 2c and “Methods”, similar results are found for the Pearson correlation, *r*). We found that the model-model *r*′ distance correlation has a similar distribution and, additionally, the distribution of correlations between model and experimental single-molecule distance matrices (average *r*′ = 0.22) is not statistically distinguishable from the one between experiments (Fig. 2c, Mann–Whitney *p* value = 0.19).

Those results show that the features of the 3D structures predicted by our model are similar to those observed in single-cell experiments, to the point that single-molecules from the model are statistically indistinguishable from experimental single-cell structures. Finally, we implemented our modeling and all the above analyses in another 2 Mb locus (chr21:28–30 Mb) investigated in human IMR90 cells by super-resolution imaging experiments^37^ and found analogous results (Supplementary Figs. 3, 4, 7, 9, 10).

The overall agreement between single-cell imaging data and the independently derived model conformations supports the view whereby, in the studied HCT116 and IMR90 loci, chromatin folding is explained at the single-cell level by a thermodynamics mechanism of globule phase separation, driven by the interactions of a few different types of binding sites, non-trivially arranged along the genome and each associated to specific combinations of chromatin organizing factors, including, but not limited to CTCF (Fig. 2a). Within that framework, the broad variability of single-molecule 3D globular structures, reflected in the varying locations of TAD-like domain boundaries, naturally results from the inherent folding degeneracy of the phase separated conformations, enhanced by the overlapping genomic organization of the different binding domains. Whereas CTCF sites are distributed over the entire locus, the boundary preferential positions correspond to the location of the edges between binding domains (Fig. 2a) as they are prone to fold in separated globules.

### Cohesin depletion reverses phase separation

**Fig. 3:**
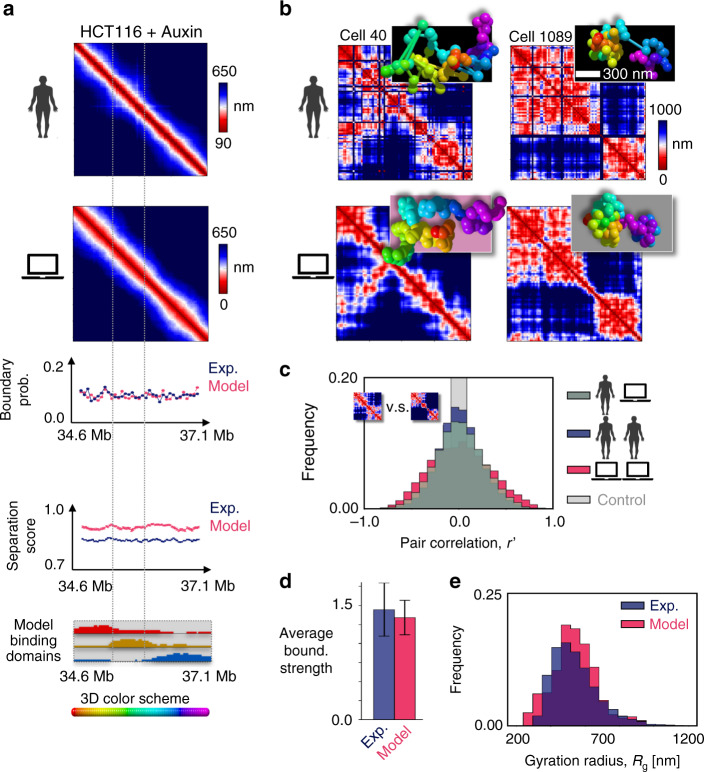
Cohesin depletion tends to reverse phase separation. **a** Top: In cohesin depleted HCT116 cells treated with Auxin (HCT116+ Auxin), the model predicted median distance matrix of the considered locus chr21:34.6–37.1 Mb also compares well against independent imaging data^37^; the Pearson and genomic-distance corrected correlations are, respectively, *r* = 0.96 and *r*′ = 0.57. A mixture of model 3D structures is required, however, 80% in the coil and 20% in the globule state. Middle: The flat domain boundary probability and separation score reflect the absence of TAD-like structures in the median matrices. Bottom: The model of the locus in cohesin depleted cells has three binding domains; their different colors are assigned by their genomic overlap with the wild-type domains of Fig. 2a. **b** Consistently, the RMSD based all-against-all comparison of single-cell^37^ (top) and model predicted (bottom) 3D structures shows that imaged conformations correspond to model structures belonging 80% to the coil (bottom left) and 20% to the globule state (bottom right, see text and Supplementary Figs. 5c, 11). The distance matrix of single molecules has non-trivial patterns in both states, but in the coil state (bottom left) contacts originate from random collisions rather than stable phase separated globule domains (bottom right). **c** The distribution of genomic-distance corrected correlations between distance matrices from single-cells (blue) is broader than in wild-type and its average is *r*′ = 0.0, highlighting a higher variability; it is statistically not distinguishable from the correlations between imaged and model distance matrices (dark gray, two-sided Mann–Whitney *p* value = 0.48). **d** In model and experiment the average boundary strength is similar (error bars s.d.), and similar to wild-type (Fig. 2d), and **e** the gyration radius distributions are not distinguishable too (two-sided Mann–Whitney *p* value = 0.10) and have a higher average value than wild-type (540 nm vs. 440 nm of Fig. 2e). The model-experiment agreement points out that cohesin depletion reverses phase separation in most cells as their corresponding model single-molecule structures are mainly in the coil rather than in the globule state, contrary to HCT116 (Fig. 2).

To investigate how acute cohesin depletion impacts single-molecule chromatin conformations, we considered the same locus in HCT116 Auxin treated cells (HCT116+ Auxin)^37^. We inferred the new SBS polymer binding sites, as before, from Hi-C data in HCT116+ Auxin cells^16^ and derived by MD the model 3D conformations to be compared with imaging data in the new cells^37^. Interestingly, in this case our approach finds only three types of specific binding sites in the locus (Fig. 3a). The domain strongly correlated with cohesin in WT HCT116 cells (green, Fig. 2a) disappears, whereas the other WT domains are overall maintained at their genomic locations, although weakened and shrunk, and their epigenetic signatures partially preserved (Fig. 3a and Supplementary Fig. 3b). We find that the new polymer model also undergoes a phase transition from a coil to a globule phase separated state, yet at around 400 nmol/l if the same affinities of the HCT116 case study model are used (Supplementary Fig. 2).

The Hi-C map of the cohesin depleted locus lacks the WT TAD-like structures and retains only a faint pattern of interactions^16^. The model recapitulates well those data too (*r* = 0.93, *r*′ = 0.33, Supplementary Fig. 4b), but we find that a mixture of 3D structures is required, composed 80% of single-molecule 3D conformations in the coil and 20% in the globule phase separated thermodynamics state. Consistently, in the HCT116+ Auxin case by the least RMSD method we find that 80% experimental structures from independent imaging data^37^ (Fig. 3b) map onto model conformations in the coil and 20% in the globule state (Supplementary Fig. 5c) in a statistically significant association (Supplementary Fig. 11). Again, the comparison of our mixture model prediction on the median distance matrix against the independent imaging data^37^ gives high correlations (*r* = 0.96, *r*′ = 0.57, Fig. 3a).

Upon cohesin depletion, although the population-averaged distance map is as featureless as the Hi-C map, in single-cell imaging data contact patterns persist, including TAD-like structures in some instances (Fig. 3b). The domain boundary strength and the average number of boundaries are similar to WT^37^. However, the imaged single-cell 3D conformations have a higher variability than WT ones: the average distance-corrected correlation, *r*′, between pairs of distance matrices is *r*′ = 0.0 and its distribution is broader (Fig. 3c). The model single-molecule conformations have also a high variability and resemble the experimental structures (Fig. 3b). Again, they have an *r*′ correlation distribution with imaged distance matrices (and with each other, average respectively *r*′ = 0.0 and *r*′ = 0.0) statistically similar to the one between experiment pairs (Mann-Whitney *p* value = 0.48, Fig. 3c). The 3D conformations of the model mixture include globular states as in WT (Fig. 3b right), but 80% of single-molecules are in the coil state (Fig. 3b left) whose contact patterns reflect transient, random chromatin collisions rather than more stably folded contacts as in WT (see “Time dynamics” section below). Consistent with such a picture, the average separation score is flat along the locus in both model and experiment (Fig. 3a). The model domain boundary probability along the locus is also as flat as the experimental one (Fig. 3a) with a similar average boundary strength (Fig. 3d); and similar are the average boundary probability and the boundary strength distribution (Supplementary Fig. 7c, d), as much as the gyration radius distribution (Mann-Whitney *p* value = 0.10 Fig. 3e), whose average value is 23% larger than in the WT case (540 nm vs. 440 nm) showing that the locus is more open.

The overall agreement between model and independent microscopy data in the HCT116+ Auxin case depicts a scenario where, consistent with the known role of cohesin as a key architecture organizing factor, cohesin depletion reverses chromatin globule phase separation to the coil thermodynamics state in single cells, whose diverse contact patterns originate mainly from random chromatin collisions rather than from phase separated domains.

### Single-molecule time dynamics

**Fig. 4:**
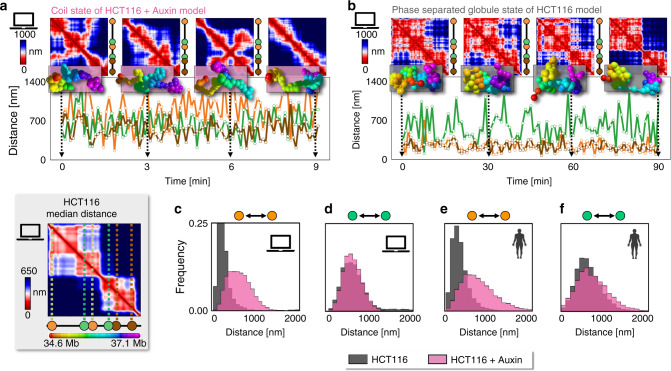
The time dynamics of single molecules illustrates how globules establish specific contacts and boundaries. The steady-state time behavior is shown of a single-molecule and its distance matrix in the model of the chr21:34.6–37.1 Mb locus in cohesin depleted (HCT116+ Auxin coil state, **a**) and wild-type cells (HCT116 globule state, **b**). The relative distances are also plotted of: (i) a pair of sites (orange), 1.2 Mb apart, in different subTADs, having a strong point-wise (loop) interaction in WT HCT116; (ii) a pair of 0.6 Mb distant sites (green) with a TAD boundary in between; (iii) a pair of sites (brown), almost 0.6 Mb apart within the same subTAD. **a** In the coil state, the distance time tracks of those sites have all wide fluctuations, as contact patterns are fleeting. **b** In the globule phase separated state, the interaction pattern shows that globules vary, but persist in time (see text). Hence, within their stochastic environment, close, specific contacts are enhanced between site pairs sharing abundant cognate binding sites (orange and brown, Supplementary Fig. 13), whereas pairs in different globules remain insulated (green). **c** The orange pair is on average much closer (Supplementary Table I) and its distance distribution much narrower in the HCT116 than in the HCT116+ Auxin model, **d** whereas the green pair behaves similarly in both. **e, f** The model distributions are close to the corresponding experimental ones^37^ in HCT116 and in HCT116+ Auxin cells, consistent with the view that chromatin folds in different thermodynamics states in WT and cohesin depleted cells.

Next, we investigated how the spatial conformations of single DNA molecules change in time and how specific patterns of contact or insulation are established, which can be uniquely achieved within our model. In the steady-state, the 3D structure of a single-molecule varies and breathes under thermal fluctuations in both the coil and phase separated states, but important differences mark the two phases (Fig. 4).

In the coil state, the contacts visible in the distance matrix of a single molecule have a highly transient nature and their pattern fully changes in time (Fig. 4a, HCT116+ Auxin model), as signaled by the average value of the *r*′ correlation between different time points that approaches zero for large time separations (Supplementary Fig. 12a), consistent with the zero average correlation between different replicates discussed before. In the phase separated state, the 3D structure also varies in time, but the long-time average *r*′ correlation remains well above zero (in the HCT116 model *r*′ plateaus to 0.39, Supplementary Fig. 12b), showing that the folded globules change, but persist in time (Fig. 4b), again consistent with the average non-zero correlation between replicates. The conformation average decay time (i.e., the time for correlations to plateau) is almost one order of magnitude larger in the globule state than in the coil state; its scale can be roughly guessed by using estimates of the viscosity of the nuclear medium reported in the literature^45,56^: for example, it results to be 9 s and 60 s respectively in the coil state of the HCT116 + Auxin and in the phase separated state of the HCT116 model (Supplementary Fig. 12).

Finally, we explored how domain boundaries and specific contact loops are established at the single-molecule level, in the face of a varying environment, by the formation of globules. To that aim, we investigated the relative distances of a particular set of sites: (i) a pair of sites (orange, Fig. 4) having in HCT116 cells a strong point-wise (loop) interaction in bulk Hi-C data, albeit located 1.2 Mb apart from each other in different subTADs; (ii) a pair of 0.6 Mb distant sites (green) with a strong TAD boundary in between; (iii) a control pair of sites (brown), almost 0.6 Mb apart, enclosed within a subTAD.

In the HCT116+ Auxin model, where molecules are mostly in the coil state, the average physical distances of the green and brown pair are comparable to each other (around 620 nm, Supplementary Table 1) and the orange pair is more open (660 nm) for its larger genomic separation. The distance distributions are comparatively broad and similar across the three pairs (Fig. 4c, d and Supplementary Fig. 13a). The situation drastically changes in the globule phase separated state of the model of HCT116 cells as the average distance of the orange and of the brown pairs is reduced of factor 2.5 down to around 280 nm. That occurs because the orange (and brown) genomic regions are enriched with cognate binding sites, which in their globule compact environment are highly likely to be bridged hence resulting in a loop visible in Hi-C bulk data. Conversely, the green sites tend to become trapped each in a different globule, remaining at roughly their coil-state distance. In this way, globules form an insulating “boundary” between them. The distance distribution of the orange (and brown) pair is much narrower in the HCT116 than in HCT116+ Auxin case, whereas the distribution of the green pair is similar in both (Fig. 4c, d and Supplementary Figs. 13a, 14a–c).

We performed an initial validation of the model time behavior by comparing the predicted distance distributions of the mentioned site pairs with single-cell imaging data, although a full test would need experiments following in real time the entire chromatin locus. Interestingly, considering the basic character of the model, its predicted distributions are comparatively close to the experimental ones, albeit there are no free parameters available in the comparison (Fig. 4e, f and Supplementary Fig. 13b). That is consistent with the above interpretation that chromatin folds in different thermodynamics states in WT and cohesin depleted cells. Finally, the time tracks (Fig. 4a, b) also clarify that the distances of all site pairs change in time subject to thermal fluctuations and, in particular, the strong point-wise loop interaction of the orange pair visible in the median distance matrix in HCT116 cells does not reflect a fixed-length permanent contact. Again, analogous results are found for the locus in IMR90 cells.

Overall, the analysis of the steady-state time dynamics shows that, while in the coil state contacts within a single molecule are fleeting and variable, in the phase separated state globules breathe and rearrange, but persist in time, as discussed in polymer physics^53^. Hence, globules can create spatially compact environments, visible as TADs and sub-TADs in Hi-C data, where specific contacts (e.g., the loops of the brown and orange pairs) are enhanced between regions sharing abundant cognate binding sites, albeit based on weak biochemical interactions. Globule boundaries also change in time, but they can efficiently separate neighboring regions along the sequence (see, e.g., the green pair), although specific contacts across proximal globules can also form (e.g., the loop of the orange pair).

## Discussion

DNA loop-extrusion has recently emerged as an important mechanism of chromatin organization^13–15^. It envisages that a cohesin complex acts as an active motor extruding loops between CTCF anchor points, in a non-equilibrium process requiring energy influx to work, e.g., ATP molecule consumption. The key role of CTCF/cohesin in chromatin architecture has been confirmed, for example, by bulk Hi-C data in systems depleted for those factors^16–18^. However, in the 2 Mb-wide loci in human HCT116 and IMR90 cells considered here, super-resolution single-cell imaging experiments hinted that DNA interactions could arise from a distinct molecular process^37^. Here, we discussed a mechanism of chromatin folding, different from the loop-extrusion, that is based on the thermodynamics of polymer phase separation and is consistent with both Hi-C and single-cell imaging data.

**Fig. 5:**
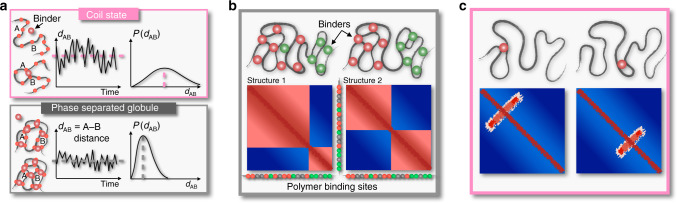
Polymer phase separation explains chromatin structure variability across single-cells. **a** In the SBS model, the polymer folds in conformational classes associated to the thermodynamics phases of the system, e.g., the coil and the phase separated globule state, according to the abundance or affinity of binding molecules. The summary cartoon highlights that in the coil phase (top) chromatin contacts tend to be more fleeting and average distances more open than in the phase separated globule state (bottom). Those thermodynamics states recapitulate single-cell architecture data. **b** Globule phase separation, for example, produces local compact environments, insulated one from the other, where specific contacts are highly enhanced between regions enriched for cognate binding sites. The inherent thermodynamic degeneracy of conformations results in a variety of single-molecule 3D structures, reflected in the variability of the genomic position of TAD-like patterns, consistent with imaging data of loci in human HCT116 and IMR90 cells. **c** Cohesin depletion, as in HCT116+ Auxin cells, tends to reverse globule phase separation into the coil state resulting in much more variable contacts, abolishing patterns in bulk Hi-C data.

Specifically, we considered a schematic polymer model of chromatin, the Strings and Binders model^19,20^, where contacts between distal binding sites are mediated by diffusing cognate bridging molecules (but our results also hold if DNA sites have direct physical interactions rather than mediated by binders). The genomic arrangements of the model putative binding sites are learned from Hi-C bulk data^6,16^ of the loci of interest, and the thermodynamics 3D conformations of the system derived from physics. Upon increasing the binder concentration, or affinity, the model undergoes a phase transition from a coil to a globule phase separated state where compact globules self-assemble by the interactions with their cognate binders. Importantly, as dictated by polymer physics^53^, the model 3D structures spontaneously fall in the conformational class corresponding to its thermodynamics phase, i.e., the coil or globule state (Fig. 5a). The consistent agreement between the predicted structures and independent single-cell super-resolution microscopy data^37^ provides evidence that, in the studied loci, chromatin folding is driven at the single-molecule level by such a mechanism of polymer phase separation.

The emerging scenario shows that in WT cells the loci fold mostly in the globule phase separated state, whose intrinsic thermodynamics degeneracy is manifested in the varying genomic positions of TAD-like patterns across single-molecules and in time (Fig. 5b). Population-averaged contact maps, such as Hi-C bulk data, capture ensemble averages and their TADs match the location of the globules that more frequently form. The analysis of the time dynamics of single molecules illustrates the diverse modes of action of globules in shaping spatial interactions or insulation between distal sites. While segregating neighboring regions, they create stable, compact local environments enhancing specific contacts between sites enriched for cognate binding sites, within and less frequently across sub-TADs and TADs. That explains how the observed stochasticity of DNA interactions, typical of weak biochemical affinities, can coexist with specificity, providing a quantitative picture on how contacts, e.g., between genes and distal regulators can be controlled at the molecular level. Finally, our results are consistent with a scenario where acute cohesin depletion tends to reverse globule phase separation into the coil state in most cells, resulting in much more variable and transient contact patterns in single molecules (Fig. 5c), hence abolishing population-averaged TAD-like domains. We find that the model inferred binding site types have significant correlations each with a specific combination of chromatin architecture factors, rather than a single one, including CTCF, Smc1, H3K27ac, or H3K27me3. That strengthens the view that the combinatorial action of different molecules, modulating each other activity, shapes the 3D architecture of the genome.

We explored a minimal model of strings and binders, but a huge diversity of microphase and phase separated structures, well beyond TAD or pled-like patterns, can be achieved by adding molecular parameters to the system^54,55^, although whether true equilibrium self-assembly can be reached in such complex systems remains to be clarified. In addition, in different chromosomal regions different physical processes could contribute or co-exist to define the architecture. Nevertheless, an organizational mechanism based on phase transitions has the advantage to be a robust and reversible procedure to trigger conformational changes: the system only needs, e.g., to establish an above threshold concentration of binders (or affinity), with no need of fine tuning their number (or strength)^19^. And phase transitions occur spontaneously sustained by the thermal bath. That could explain how simple cell strategies of upregulation of genes associated to transcription factors or epigenetic modifications can reliably shape the self-assembly of chromatin architectures in the nucleus.

## Methods

### Loci and datasets

The coordinates of studied 2.5 Mb-wide locus in human WT HCT116 and cohesin depleted HCT116+ Auxin cells are chr21:34600000–37100000 (hg38), those of the locus in IMR90 cells are chr21:28000000–30000000. For those regions, published single-cell imaging data at 30 kb resolution were taken from^37^. Published independent in situ Hi-C data in HCT116, HCT116+ Auxin cells were taken from^16^ and in IMR90 cells from^6^. We employed KR normalized^57^, 5 kb resolution Hi-C data, re-binned at 30 kb by summation as in^37^.

### The strings and binders (SBS) polymer model

To investigate the 3D folding of the considered loci we used the Strings and Binders (SBS) polymer model of chromatin^19,20^. In the SBS model, a chromatin region is represented as a Self-Avoiding Walk (SAW) chain of beads, having different specific types of binding sites for cognate diffusing molecular binders. Each different type is visually represented by a different color in our notation. In our model a specific attractive interaction is only allowed between polymer beads and binders of the same color. We also considered the case where along the chain there are unspecific binding sites for binders, characterized by a lower affinity (see “Molecular Dynamics” section below). In addition, we explored a variant of the model where direct interactions between cognate DNA sites are used, rather than mediated by binders, and our conclusions remain unchanged, as expected from Statistical Mechanics. To derive the SBS model of a locus of interest, i.e., the minimal number of distinct types of specific binding sites and their positioning along the polymer chain, we developed a machine learning procedure based on our previously published PRISMR method^50^. As explained below, the procedure returned polymer models made of 830 beads in HCT116, with four different binding types in WT (Fig. 1) and three distinct binding domains in cohesin depleted cells (Fig. 3). In IMR90 cells the model has 650 beads with seven different types of sites (Supplementary Fig. 9). Once derived the optimal polymer models for our genomic loci, we performed massive parallel MD simulations^47^ to produce an ensemble of single-molecule conformations at thermodynamic equilibrium.

### Machine learning the polymer models of the studied loci

In this study we developed an improved machine learning procedure to infer the minimal, best polymer model for a given genomic locus, based on our previously published PRISMR method^50^. For sake of clarity, we first summarize the main points of the original PRISMR method and next discuss the important developments here implemented to improve it.

The PRISMR procedure infers the best, minimal SBS polymer model of a given genomic locus starting from only its corresponding bulk Hi-C data. It finds the minimal arrangement of binding sites on the polymer chain that best reproduces the input Hi-C matrix based only on physics. To take into account the possibility of multiple binding sites within a DNA window at the considered Hi-C data resolution (here 30 kb), we suppose that our model can accommodate up to *r* binding sites (beads) in each DNA window. Hence, the total number of beads in the SBS model chain of the considered locus is the product of the number of DNA windows of the locus times *r*. The procedure also estimates the optimal value of *r*. For the loci considered in this study, we used 30 kb resolution Hi-C data and found that the optimal value of *r*, r^*^ is ≤10 (see below).

PRISMR is based on a Simulated Annealing Monte Carlo (SA) procedure that minimizes a cost function *H*_0_, chosen to be the standard mean squared error function, i.e., the average squared distance between the input and model derived contact matrix. In order to reduce overfitting, the cost function also includes an additional Bayesian term (a chemical potential), *H*_λ_, that penalizes the addition of new interacting beads. The Bayesian term is weighted by a regularization parameter *λ* ≥ 0, which sets the cost of adding a single new interacting binding site to the polymer model. PRISMR seeks the minimum of the total cost function, *H*, in the space of all SBS polymers with *n* allowed colors by the SA iterative procedure: at each iteration the color (type) of a randomly chosen bead is changed at random, the contact matrix of the new polymer is computed out of physics, and the cost function computed until convergence. The procedure is repeated many times using different initial conditions to scan the space of the parameters *n* and *λ*, in order to find the optimal values of n^*^ and λ^*^ to explain the input Hi-C contact matrix within a given accuracy^50^.

In this study, we changed and redesigned different aspects of the PRISMR algorithm. First, to better take into account genomic distance effects within our procedure, we implemented a new cost function. In brief, that is achieved by scaling each term of the mean squared error *H*_0_ by the average Hi-C intensity at the corresponding genomic distance. That improves the method performances at larger genomic separations as it prevents the data close to the diagonal of the Hi-C matrix dominating the calculations due to their much higher values with respect to those corresponding to large genomic separations.

Next, we improved the method to estimate the optimal number n^*^ of different types of binding sites of the putative polymer model of the genomic locus of interest. That is a crucial parameter, as it corresponds to the number of predicted different types of binding molecular factors that give rise to the locus contact matrix. To this aim, in an approach standard to supervised learning, we split our Hi-C dataset in two complementary sets: a training set and a test set (Supplementary Fig. 1a). PRISMR is run on the training set, i.e., in the SA procedure the cost function is evaluated only on those matrix elements. In all the cases discussed here, we split randomly the Hi-C data into a 70% training set and a 30% test set. However, we checked that the estimated model parameters are robust by varying the training set size from 50 to 80% of the Hi-C data. To estimate the best number of colors, n^*^, the SA procedure is repeated for different values of *n*, and the cost function is evaluated for the output models, both on the training and test sets. For each value of *n*, we ran at least 20 independent SA simulations with varying initial conditions, i.e., with different random initializations of the polymer model, and with different random selections of the training dataset. Supplementary Fig. 1b, c shows the cost function minimum *H*_0_(*n*) as function of *n*, for the HCT116 and HCT116+ Auxin loci, normalized by its value for a polymer having no binding sites, *H*_0_(*n* = 0). As expected, the cost function evaluated over the training set decreases with *n*, toward an asymptotic plateau, as previously found^50^, so that the agreement between the experimental data and the theoretical model improves more and more by increasing *n*. Conversely, the cost function evaluated over the test set first decreases with *n* up to reach a minimum and then it increases, signaling that overfitting sets in. The value of *n* corresponding to the minimum of *H*_0_ over the test set is the sought optimum n^*^, for which the model has the best predictive power. Such a procedure allows thus to identify in a clear, quantitative way the optimal number of different binding site types in the model. It returns n^*^ = 4 in HCT116, n^*^ = 3 in HCT116+ Auxin and n^*^ = 7 in IMR90. In order to further reduce overfitting, for a given *n*, the cost function of our procedure also includes the regularization term *H*_λ_ discussed above, which penalizes the addition of colored beads. To find the optimal *λ* value, *λ* ^*^, we fixed n^*^ and minimized the total cost function *H* = *H*_0_ + *H*_λ_ at varying values of *λ*, so to find *λ* ^*^ as the minimum of *H*_0_ in the test set. We proceeded as for the estimation of n^*^. Precisely, we split Hi-C data in a 70% training and a 30% test data and performed the PRISMR optimization only on the training set. We ran at least 20 independent simulations with varying initial conditions and evaluated the optimal λ^*^ as the value for which the minimum of *H*_0_ over the test set is attained. That returns λ^*^ = 10^−5^ in HCT116 and HCT116 + Auxin and λ^*^ = 10^−4^ in IMR90. Next, we fixed n^*^and λ^*^ and proceeded to estimate the minimal number of polymer beads per 30 kb window, r^*^, required to explain data within a given accuracy, as done in^50^. We find r^*^ values ranging from 7 to 10 in the considered loci and for simplicity we set r^*^ = 10 in all cases. Finally, by using the estimated optimal parameters n^*^, r^*^, λ^*^, we ran an additional battery of up to 5 × 10^2^ independent SA simulations from different initial conditions to identify the final output of the procedure, i.e., the polymer model corresponding to the absolute minimum of the cost function (Figs. 2a, 3a and Supplementary Fig. 9a, bottom panels). As discussed in^50^, the models corresponding to the lower 10% minima are consistently similar to each other, showing the robustness of the procedure.

### Correlation of model binding domains with epigenetic data

We compared the model inferred binding domains with a set of epigenetic tracks available in the studied cell types (Supplementary Fig. 3a–c). In HCT116 cells we used Chip-seq data available from^16^ (GEO accession: GSM2809609, GSM2809611, GSM2809613, GSM2809617–30) and from the ENCODE database^58^ (ENCODE accession: ENCFF175RBN, ENCFF001UDL, ENCFF001UDN, ENCFF001UDP, ENCFF001UDT, ENCFF001UDV, ENCFF001UDX, ENCFF001UEB, ENCFF001UED, ENCFF001UEJ, ENCFF001UEL, ENCFF001UEN, ENCFF001UEP, ENCFF001UER, ENCFF088WYS, ENCFF144BSH, ENCFF617QEN). In IMR90 we used data from ENCODE (ENCFF195CYT, ENCFF116RLU, ENCFF453XKM, ENCFF899APS, ENCFF474OJM, ENCFF752IXO, ENCFF178QVF, ENCFF741WIY, ENCFF625BTD). After binning the epigenetic tracks at 30 kb resolution, we calculated the Pearson correlation coefficient between each binding domain—epigenetic mark pair, in the considered loci. To test the statistical significance of the obtained correlations, we compared them with a random control model. The control correlation distribution was built by computing correlations between the above chromatin marks and randomized binding domains (10^3^ different realizations for each case), obtained by bootstrapping their binding sites positioning^50^. We then considered positive correlations significant if above the 90th percentile and negative correlations significant if below the 10th percentile of the random control distribution. The resulting significant correlations are represented in the heatmaps of Supplementary Fig. 3a, b, c.

### Molecular dynamics simulations

Our polymer system is subject to a Langevin dynamics, numerically solved using the Verlet algorithm within the LAMMPS package^59^. Its interactions potentials are taken from classical polymer physics studies^60^ and detailed in^47^. The initial states of our MD simulations are distinct open SAW conformations. The binders also move under the Langevin equation within the simulation box (which has periodic boundary conditions) and interact with the specific and unspecific polymer binding sites, so driving the folding of the chain. We let the system evolve up to when stationarity is reached, as shown by the plateauing of the gyration radius and binding energy as function of the MD time iteration steps (Supplementary Fig. 2a). The features of our MD simulations and all details are discussed in^47^. In our simulations we computationally sampled a range of specific and unspecific binding energy affinities, in the weak biochemical energy range, respectively from 3.1 to 8.0*K*_B_*T* and from 0 to 2.7*K*_B_*T* (*K*_B_ is the Boltzmann constant and *T* is the system temperature in Kelvin). For sake of simplicity, we kept the affinities equal respectively across the different specific and across the unspecific binding sites. The dimensionless parameters of our MD simulations are converted into physical units via the standard MD procedure^61,62^. The length scale of the model, i.e., the bead diameter *σ*, is calibrated by equating the medians of the model and experimental^37^ gyration radius distributions (Figs. 2e, 3e and Supplementary Fig. 9e). We find *σ* = 45 nm in the HCT116, *σ* = 22 nm in HCT116+ Auxin, and *σ* = 60 nm in IMR90 model. In the HCT116+ Auxin modeling, where a coil-globule mixture of model conformations best explains the experimental data, as an additional exercise we also tried to estimate independently the length scales, *σ*’s, of the structures in the two thermodynamic phases, as done for instance in Fig. 4c. The MD time scale, *τ*, is *τ* = 6π*ησ*^3^/(*K*_B_*T*), where *η* is the solvent viscosity. Reference values of the nucleoplasm viscosity range around *η* = 0.03*P*^45,56^, which we use here. Changes to the viscosity proportionally change the time scale. The molar binder concentration, *c*, is *c* = *P*/(*VN*_A_), where *P* is the total number of binders in the simulation cubic box, *V* is the box volume (whose linear size is taken to be equal to the gyration radius of a SAW polymer with a corresponding number of beads), and *N*_A_ is the Avogadro number. We explored with our MD simulations almost three orders of magnitude in binder concentrations for each of the HCT116, HCT116+ Auxin and IMR90 models (Fig. 1c, Supplementary Fig. 2a, b). For example, our case study concentration for the globule phase separated state (see next section) is: *c* = 0.11 μmol/l in HCT116, *c* = 0.78 μmol/l in HCT116+ Auxin, *c* = 0.05 μmol/l in IMR90. Analogously, the case study coil state concentration is: *c* = 0.01 μmol/l in HCT116, *c* = 0.08 μmol/l in HCT116+ Auxin, *c* = 0.007 μmol/l in IMR90. For each studied locus and for each considered binder concentration and affinity, we produced a statistical ensemble of 1000 distinct equilibrium single-molecule 3D configurations by massive MD simulations as described above. The POV-RAY software (Persistence of Vision Pty. Ltd., 2004) is used to produce the plots of the 3D experimental^37^ and model conformations. The conformation spatial coordinates are interpolated by simple linear splines.

### System order parameters and phase transition

The SBS polymer models of each of the studied genomic loci undergo, upon increasing the binder concentration or affinity, a phase transition from a coil to a more compact, globule phase separated state, as signaled by the system order parameters, the gyration radius (or binding energy) and the average separation score (see sections below for definitions and computational details). Figure 1c (top) and Supplementary Fig. 2b (top), corresponding respectively to HCT116 and HCT116+ Auxin, show the equilibrium value of the polymer gyration radius normalized respect to its SAW value at increasing binder concentrations. In both cases, a sharp drop of the gyration radius occurs at a characteristic concentration threshold, around 50 nmol/l in HCT116 and 400 nmol/l in HCT116+ Auxin for the case study affinity considered in the Main Text. At the same threshold a drop is also found for the system binding energy (Supplementary Fig. 2a, b), i.e., the total potential energy of the simulated system. Analogously, at the same threshold the system average separation score^37^ drops (Fig. 1c, Supplementary Fig. 2b). The separation score measures the level of spatial separation between chromatin segments on either side of a given genomic position. Its sharp decrease signals that, when the number of binders (or their affinity) increases above threshold, distinct spatially segregated globules self-assemble along the polymer chain. As known in polymer physics^53^, the simultaneous sharp drop of the gyration radius (and binding energy) and separation score signals the phase transition of the system from the coil state, where the polymer is in randomly folded conformations, to a globule state in which the polymer, due to attractive interactions, forms more compact, separated globules. A similar transition occurs in the IMR90 model. By the least RMSD method (Supplementary Fig. 5, see “Structural comparison of experimental and model 3D conformations by RMSD” section below), we find in the HCT116 case that 100% of experimental structures are mapped in a statistically significant association onto conformations of the model belonging to the thermodynamics globule states. Similarly, 99% of experimental structures in IMR90 become mapped onto model conformations in the globule state and 1% in the coil state. In the case of HCT116+ Auxin cells, 80% experimental structures map onto model 3D conformations in the coil and 20% in the globule state.

### Contact frequency matrices and correlations

To compute the model average pairwise contact matrix, we adopted a standard method used in the literature^45,50^. Briefly, for each polymer 3D conformation, we consider two sites in contact if their relative Euclidean distance is less than a threshold *Aσ*, where *A* is a dimensionless constant. The model-predicted average (or median) matrix is simply the average (or median) of the single-molecule matrices across the considered ensemble. We checked that by changing *A* in a window ranging from three to ten, similar results are found. In all the studied loci, we found very high Pearson correlations, *r*, between the experimental^6,16^ and the contact matrices of the mixture models: *r* = 0.88 in HCT116 (Supplementary Fig. 4a), *r* = 0.93 in HCT116+ Auxin (Supplementary Fig. 4b), and *r* = 0.94 in IMR90 (Supplementary Fig. 4c). To get a better measure of similarity, we also evaluated the genomic distance-corrected Pearson correlation coefficient, *r*′^50^. Specifically, *r*′ is the Pearson correlation computed on contact matrices where from each element the mean value of the diagonal to which it belongs to is subtracted. The *r*′ correlations between model and Hi-C bulk data are: *r*′ = 0.68 in HCT116, *r*′ = 0.33 in HCT116 + Auxin, *r*′ = 0.74 in IMR90.

### Spatial distance matrices

The single-molecule distance matrix is the matrix of all pairwise Euclidean distances between the beads of the considered polymer conformation and we computed it with the Python SciPy package. As above, the model-predicted median distance matrix is simply the median of the single-molecule distance matrices across the considered ensemble. As in the experimental paper^37^, distance matrices are represented as two-dimensional heatmaps with the seismic reversed color bar. In Fig. 3b, the color bar scale is set by use of the same percentiles in both the experimental and model matrices to have a fair comparison. In the investigated loci, the model median spatial matrices have correlation values with the experimental ones equal to: *r* = 0.95 and *r*′ = 0.84 in HCT116 (Fig. 2a), *r* = 0.96 and *r*′ = 0.57 in HCT116+ Auxin (Fig. 3a), and *r* = 0.96 and *r*′ = 0.77 in IMR90 (Supplementary Fig. 9a). Here and in the following analyses, we filtered out the experimental single-cell distance matrices^37^ having NaN values for more than 80% of the entries and, in order to remove outliers, the matrices having a Pearson correlation <0.01 with the others are also removed in both models and experiments.

### Gyration radius and separation score

We analyzed the ensemble distribution of the gyration radius in models and experiments^37^, filtering out outliers. In the three studied loci, we find that the model and experimental gyration radius distributions are not statistically distinguishable (*p* = 0.40 in HCT116, *p* = 0.10 in HCT116+ Auxin, *p* = 0.68 in IMR90, two-sided Mann–Whitney *p* value). In both the HCT116 and IMR90 loci, the experimental and model average gyration radius is 440 nm (Fig. 2e, Supplementary Fig. 9e), while in the HCT116+ Auxin case the average value increases to 540 nm (Fig. 3e).

We employed the definition of the separation score and the computational algorithm to compute it reported in^37^. We studied in our three loci the separation score as a function of the genomic coordinate, comparing the model predictions with the experimental curves and finding overall high correlations (*r* = 0.85 in HCT116, Fig. 2a; *r* = 0.41 in HCT116+ Auxin, Fig. 3a; *r* = 0.79 in IMR90, Supplementary Fig. 9a, errors represent the 95% confidence interval). Note that no free parameters are available in the calculations and in the comparisons.

### TAD boundary probability and boundary strength

To compute boundary probabilities and strengths we used the methods and algorithms discussed in^37^. The algorithm parameters used for the experimental data^37^ are: gb = 1, valley = 1, su = 10, sl = 6. We checked that small changes in those parameters do not strongly affect the results, such as the location of boundaries. For instance, in the HCT116 and HCT116 + Auxin models, we used: gb = 1, valley = 4, su = 5, sl = 5. In the IMR90 model: gb = 1, valley = 8, su = 4, sl = 4. We obtained high Pearson correlations in the comparison of the experimental and model derived boundary probability along the locus in HCT116 (Fig. 2a) and IMR90 (Supplementary Fig. 9a), respectively *r* = 0.79 and *r* = 0.60. In HCT116+ Auxin (Fig. 3a), where the boundary probability is flat because the positions of the domain boundaries fluctuate uniformly along the genomic coordinate, a lower Pearson, *r* = 0.19, is found as expected. To curate noise, we performed a two-point running average in the plots of the boundary probabilities. Importantly, in agreement with the experiments^37^, we found that the model boundary probability averaged on the genomic coordinates is comparable in HCT116 and HCT116+ Auxin and those are similar to their experimental values (Supplementary Fig. 7a, c, error bars are the standard deviation of the mean). Also in IMR90, the average boundary probability is comparable with the experimental value (Supplementary Fig. 7e) and, interestingly, it is similar to HCT116 as experimentally found. The distributions of boundary strengths for the three analyzed loci are reported in Supplementary Fig. 7b, d, f. The average model boundary strengths are also very similar to the corresponding experimental values (Figs. 2d, 3d, Supplementary Fig. 9d, bars are the standard deviation). Note that no free parameters are available in those comparisons.

### Variability of single-molecule 3D structures

To measure the degree of variability of single-molecule conformations we analyzed the distribution of the Pearson *r*′ correlations between pairs of single-molecule distance matrices from both experiments and models. Specifically, we computed (Figs. 2c, 3c, and Supplementary Fig. 9c): (a) the *r*′ correlation between all the pairs of experimental single-cell distance matrices^37^ (blue histogram, referred hereafter as exp.-exp. *r′* distribution); (b) the *r′* correlation between all the pairs of model single-molecule distance matrices (red, model-model *r*′ distribution in the following); (c) the distribution of *r*′ correlations between model and experimental single-molecule distance matrix pairs (dark gray, model-exp. *r*′ distribution); (d) the *r′* correlations in a random control case (gray), i.e., between pairs of randomized single-molecule distance matrices derived from single-cell experimental data^37^ with bootstrapped diagonals. To smooth the effects of random noise in those calculations, we applied a Gaussian filter on single-cell distance matrices, using a standard deviation of the Gaussian kernel equal to 1. We performed a two-sided Mann–Whitney test to quantify the statistical similarity between the *r*′ distributions of the different cases. Importantly, in the test we only considered independent pairs of distance matrices, selecting samples of the same size in both models and experiments. In addition, we averaged the test over ten distinct samples of independent pairs of matrices so to refine our estimate. As an example, in the comparison between the exp.–exp. and the model-exp. *r*′ distribution, we computed a Mann–Whitney *p* value > 0.01 in all three examined cases (*p* = 0.19 in HCT116, *p* = 0.48 in HCT116+ Auxin, *p* = 0.02 in IMR90), meaning that the two distributions are not statistically distinguishable from each other.

### Structural comparison of experimental and model 3D conformations by RMSD

To show that the model structures of the studied loci are a bona-fide representation of the conformational space explored in single cells, we also directly compared pairs of 3D structures from experiments and model. To this aim, we employed an accepted method that finds the optimal rotation between two centered 3D structures to minimize their coordinate difference, measured as their mean squared deviation (RMSD)^33^. In this way each 3D structure from imaging data is univocally associated to a corresponding model 3D structure by searching for the minimal RMSD (Supplementary Fig. 5a). To fairly compare 3D structures from imaging data and modeling, we normalized both experimental and model coordinates by a standard *z*-score.

In the case of the HCT116 cell locus, we found that 100% of experimental structures are mapped onto conformations of the model belonging to the thermodynamics globule state (Supplementary Fig. 5b). To test that the association is far from random, we compared the RMSD distribution of the experiment-model optimal matches to the RMSD distribution of pairwise comparisons between experimental structures (null model): the two distributions are statistically different (Mann-Whitney test *p* value = 0) with only 2% of entries of the former falling above the first quartile of the latter (Supplementary Fig. 6b). In the HCT116, as well as in the HCT116 + Auxin and IMR90 models, we also found that, by matching model to experimental conformations, the RMSD distribution is well within the distribution of RMSDs of the experiment-model optimal pairs (and below the bottom 5% of the null model), showing that each model structure is significantly similar to at least one corresponding experimental conformation. That highlights that our modeling structures are well represented in the experimental ensemble (Supplementary Fig. 6a).

In the case of HCT116+ Auxin cells we found that ~80% of experimental structures map onto model conformations in the coil (open) state and the remaining 20% onto model globule states (Supplementary Fig. 5c), confirming that a mixture of thermodynamics states describes the experimental data, consistent with our other findings (see Main Text). Note that, also in this case, best match pairs have indeed very similar distance matrices (Supplementary Fig. 11a), supporting our other method to perform an all-against-all comparison by computing distance matrix correlations. Again, to test that the above association between structures is statistically significant we compared the distribution of RMSDs of the experiment-model optimal matches to the distribution of RMSDs from pairwise comparisons between experimental structures (null model) (Supplementary Fig. 11b). The two distributions are statistically different (Mann–Whitney test *p* value = 0) and, additionally, they are well separated: only 8% of entries of the former fall above the first quartile of the latter. That highlights that our association criterion is statistically significant and far from random.

In the case of the IMR90 cell locus, we also found that 99% of experimental structures are mapped onto model conformations in the globule states (Supplementary Fig. 5d); again, each model conformation is significantly similar to at least one experimental structure (Supplementary Fig. 10a). Finally, the RMSD distribution of the experiment-model optimal matches and the RMSD distribution of pairwise comparisons between experimental structures are statistically different (Mann–Whitney test *p* value = 0) with only 2% of entries of the former falling above the first quartile of the latter (Supplementary Fig. 10b).

### Control block-copolymer model

As a comparison with our SBS model, we also considered a control block-copolymer model designed specifically to reproduce the four main TAD-like structures visible in bulk Hi-C data of the HCT116 cell locus. By construction the block-copolymer has precisely the same number of degrees of freedom of our SBS model, i.e., the same number of binding site types (colors) and of beads, but with no intertwining between them (Supplementary Fig. 8a). We used such a model as a control where to repeat all our analyses.

We found that the block-copolymer model poorly reflects the complexity of the observed contact patterns and, in particular, inter- and intra-TAD signals (Supplementary Fig. 8b). Its correlation with median imaged distance data is *r*′ = 0.54, while our model has *r*′ = 0.84. Note that the pattern of intra-TAD signals is confirmed by two independent technologies, Hi-C and super-resolution microscopy^37^, so it must be accounted for by models, as done by our SBS model. Thus, the intertwining of colors (i.e., of binding sites) in our SBS model is necessary to capture important experimental evidences, missed by the control block-copolymer model. Importantly, the arrangement of colors in our SBS model is statistically different from a random arrangement as well as from an arrangement where colors are perfectly separated as in a block-copolymer model. To assess that, we measured the overlaps^50^ of the model binding domains with each other and compared them against the overlaps found in a control random model, obtained by bootstrapping the colored binding sites positions, and in the control block-copolymer model. We found an average overlap between different colors around 50%, significantly smaller (*p* value = 1e−3, Mann-Whitney test) than the average overlap found in the random control (around 70%), and significantly higher (*p* value = 1e-3, Mann-Whitney test) than the average overlap in the block-copolymer model, which is equal to zero by construction.

As expected by construction, we found that the boundary probability of the control block-copolymer model has peaks where the SBS model and real data have peaks (Supplementary Fig. 8c), however experimental data are less well reproduced by the control (correlation *r* = 0.47) than by our model (*r* = 0.79). In particular, the control model peaks are four times higher than those from experiments and from our model, showing that the separation of the globules is much stronger in the control than in our SBS model.

Finally, we computed the pair correlation, *r*′, between the control block-copolymer model and experimental distance matrices and found that the distribution of *r*′ compares to the experimental one worse than our SBS model (Supplementary Fig. 8d). In particular, the average value of *r*′ in the block-copolymer model is 33% higher than in the experiment, showing that in the former there is a lower conformational variability.

### Steady-state dynamics and time correlations

We studied the steady-state time dynamics of single-molecule conformations. In Fig. 4a, b we plotted at different times the distance matrices and the corresponding 3D structures of a single-molecule respectively in the coil state of the case study HCT116+ Auxin model and in the globule phase separated state of the case study HCT116 model. Similar findings are obtained for the IMR90 model. To get an estimate of the conformation average decay time we measured, in the abovementioned pure states of the models, the *r*′ correlations between single-molecule distance matrices at different lag times. The time behavior of such correlation is shown in Supplementary Fig. 12, where we superimpose a stretched exponential fit. The decay time is defined as the lag time where the average *r*′ time correlation has spanned 95% of its total variation range. By using the estimate of the nuclear viscosity discussed above (see “Molecular dynamics” section), the decay time is 9, 60, and 90 s, respectively in the coil state of the HCT116+ Auxin model and in the phase separated states of the HCT116 and IMR90 models. Consistent with the ensemble correlation analysis (see “Variability of single-molecule 3D structures” above), the long-time *r*′ self-correlation approaches zero in the coil state of the HCT116+ Auxin model, while it has a non-zero value in the phase separated states of HCT116 and IMR90 (respectively 0.39 and 0.30). In the mentioned cases, we also measured the single-molecule relative distances of specific site pairs (orange, green, and brown in Fig. 4) corresponding to the following genomic coordinates in HCT116 cells: orange: 34.69–35.80 Mb; green: 35.59–36.25 Mb; brown: 36.43–36.91 Mb. In Fig. 4c–f and Supplementary Fig. 13a, b we computed the mixture model ensemble distance distributions of those pairs and compared them with the corresponding experimental ensemble distributions^37^. We also performed the same calculation in the HCT116+ Auxin simulated polymers using only the coil pure state, as shown in Supplementary Fig. 14a–c. In our analysis, to correct for outliers, we did not consider distances above 2000 nm. Average values and standard deviations of the measured distance distributions are computed for computational reasons on a random sample of all model conformations and summarized in Supplementary Table I.

### Reporting summary

Further information on experimental design is available in the Nature Research Reporting summary linked to this paper.

## Supplementary information

Supplementary Information

Reporting Summary

## Data Availability

The data supporting the findings of this study are available from the corresponding author upon request.

## Code availability

We used the publicly available LAMMPS software for the Molecular Dynamics simulations of the SBS polymer modeling. Analyses involving the computation of spatial distances were performed using built-in functions within the Python SciPy software (version 1.3.1). The structural comparison of experimental and model conformations by RMSD was performed using the free available MDAnalysis Python library. We employed the POV-Ray software (version 3.7) to produce the 3D snapshots. Custom codes used to generate results reported in the manuscript can be made available from the corresponding upon request. All details of the algorithms are illustrated in the Methods section and in previous publications cited therein.

## References

[1] Bickmore WA, Van Steensel B (2013). Genome architecture: domain organization of interphase chromosomes. Cell.

[2] Dekker J, Mirny L (2016). The 3D genome as moderator of chromosomal communication. Cell.

[3] Dixon JR, Gorkin DU, Ren B (2016). Chromatin domains: the unit of chromosome organization. Mol. Cell.

[4] Spielmann M, Lupiáñez DG, Mundlos S (2018). Structural variation in the 3D genome. Nat. Rev. Genet..

[5] Finn EH, Misteli T (2019). Molecular basis and biological function of variability in spatial genome organization. Science.

[6] Rao SSP (2014). A 3D map of the human genome at kilobase resolution reveals principles of chromatin looping. Cell.

[7] Nora EP (2012). Spatial partitioning of the regulatory landscape of the X-inactivation centre. Nature.

[8] Dixon JR (2012). Topological domains in mammalian genomes identified by analysis of chromatin interactions. Nature.

[9] Fraser J (2015). Hierarchical folding and reorganization of chromosomes are linked to transcriptional changes in cellular differentiation. Mol. Syst. Biol..

[10] Lieberman-Aiden E (2009). Comprehensive mapping of long-range interactions reveals folding principles of the human genome. Science.

[11] Beagrie RA (2017). Complex multi-enhancer contacts captured by genome architecture mapping. Nature.

[12] Quinodoz SA (2018). Higher-order inter-chromosomal hubs shape 3D genome organization in the nucleus. Cell.

[13] Sanborn AL (2015). Chromatin extrusion explains key features of loop and domain formation in wild-type and engineered genomes. Proc. Natl Acad. Sci. U.S.A..

[14] Fudenberg G (2016). Formation of chromosomal domains by loop extrusion. Cell Rep..

[15] Brackley CA (2017). Nonequilibrium chromosome looping via molecular slip links. Phys. Rev. Lett..

[16] Rao SSP (2017). Cohesin loss eliminates all loop domains. Cell.

[17] Schwarzer W (2017). Two independent modes of chromatin organization revealed by cohesin removal. Nature.

[18] Nora EP (2017). Targeted degradation of CTCF decouples local insulation of chromosome domains from genomic compartmentalization. Cell.

[19] Nicodemi M, Prisco A (2009). Thermodynamic pathways to genome spatial organization in the cell nucleus. Biophys. J..

[20] Barbieri M (2012). Complexity of chromatin folding is captured by the strings and binders switch model. Proc. Natl Acad. Sci. U. S. A..

[21] Barbieri M (2017). Active and poised promoter states drive folding of the extended HoxB locus in mouse embryonic stem cells. Nat. Struct. Mol. Biol..

[22] Shin Y, Brangwynne CP (2017). Liquid phase condensation in cell physiology and disease. Science.

[23] Hnisz D, Shrinivas K, Young RA, Chakraborty AK, Sharp PA (2017). A phase separation model for transcriptional control. Cell.

[24] Boija A (2018). Transcription factors activate genes through the phase-separation capacity of their activation domains. Cell.

[25] Cho WK (2018). Mediator and RNA polymerase II clusters associate in transcription-dependent condensates. Science.

[26] Sabari BR (2018). Coactivator condensation at super-enhancers links phase separation and gene control. Science.

[27] Chong S (2018). Imaging dynamic and selective low-complexity domain interactions that control gene transcription. Science.

[28] Guo YE (2019). Pol II phosphorylation regulates a switch between transcriptional and splicing condensates. Nature.

[29] Larson AG (2017). Liquid droplet formation by HP1α suggests a role for phase separation in heterochromatin. Nature.

[30] Strom AR (2017). Phase separation drives heterochromatin domain formation. Nature.

[31] Nagano T (2013). Single-cell Hi-C reveals cell-to-cell variability in chromosome structure. Nature.

[32] Flyamer IM (2017). Single-nucleus Hi-C reveals unique chromatin reorganization at oocyte-to-zygote transition. Nature.

[33] Stevens TJ (2017). 3D structures of individual mammalian genomes studied by single-cell Hi-C. Nature.

[34] Nagano T (2017). Cell-cycle dynamics of chromosomal organization at single-cell resolution. Nature.

[35] Boettiger AN (2016). Super-resolution imaging reveals distinct chromatin folding for different epigenetic states. Nature.

[36] Cattoni DI (2017). Single-cell absolute contact probability detection reveals chromosomes are organized by multiple low-frequency yet specific interactions. Nat. Commun..

[37] Bintu B (2018). Super-resolution chromatin tracing reveals domains and cooperative interactions in single cells. Science.

[38] Cardozo Gizzi AM (2019). Microscopy-based chromosome conformation capture enables simultaneous visualization of genome organization and transcription in intact organisms. Mol. Cell.

[39] Finn EH (2019). Extensive heterogeneity and intrinsic variation in spatial genome organization. Cell.

[40] Li Q (2017). The three-dimensional genome organization of Drosophila melanogaster through data integration. Genome Biol..

[41] Serra F (2017). Automatic analysis and 3D-modelling of Hi-C data using TADbit reveals structural features of the fly chromatin colors. PLoS Comput. Biol.

[42] Nir G (2018). Walking along chromosomes with super-resolution imaging, contact maps, and integrative modeling. PLoS Genet..

[43] Lin D, Bonora G, Yardimci GG, Noble WS (2018). Computational methods for analyzing and modeling genome structure and organization. Wiley Interdiscip. Rev. Syst. Biol. Med.

[44] Bohn M, Heermann DW (2010). Diffusion-driven looping provides a consistent provides a consistent framework for chromatin organization. PLoS One.

[45] Brackley CA, Taylor S, Papantonis A, Cook PR, Marenduzzo D (2013). Nonspecific bridging-induced attraction drives clustering of DNA-binding proteins and genome organization. Proc. Natl Acad. Sci..

[46] Jost D, Carrivain P, Cavalli G, Vaillant C (2014). Modeling epigenome folding: formation and dynamics of topologically associated chromatin domains. Nucleic Acids Res..

[47] Chiariello AM, Annunziatella C, Bianco S, Esposito A, Nicodemi M (2016). Polymer physics of chromosome large-scale 3D organisation. Sci. Rep..

[48] Di Pierro M, Zhang B, Aiden EL, Wolynes PG, Onuchic JN (2016). Transferable model for chromosome architecture. Proc. Natl Acad. Sci. U.S.A..

[49] Tjong, H. et al. Population-based 3D genome structure analysis reveals driving forces in spatial genome organization. *Proc. Natl. Acad. Sci. U.S.A*. 10.1073/pnas.1512577113 (2016). 10.1073/pnas.1512577113 PMC4812752 26951677

[50] Bianco S (2018). Polymer physics predicts the effects of structural variants on chromatin architecture. Nat. Genet..

[51] Buckle A, Brackley CA, Boyle S, Marenduzzo D, Gilbert N (2018). Polymer simulations of heteromorphic chromatin predict the 3D folding of complex genomic loci. Mol. Cell.

[52] Shi G, Liu L, Hyeon C, Thirumalai D (2018). Interphase human chromosome exhibits out of equilibrium glassy dynamics. Nat. Commun..

[53] De Gennes, P. G. Scaling Concepts in Polymer Physics. (Cornell University Press, Ithaca N.Y., 1979) 10.1163/_q3_SIM_00374.

[54] Bates, F. S. & Fredrickson, G. H. Block copolymers-designer soft materials. *Phys. Today*, 10.1063/1.882522 (1999).

[55] Hamley I.W. *The Physics of Block Copolymers*. (Oxford University Press, 1999).

[56] Baum M, Erdel F, Wachsmuth M, Rippe K (2014). Retrieving the intracellular topology from multi-scale protein mobility mapping in living cells. Nat. Commun..

[57] Knight, P. A. & Ruiz, D. A fast algorithm for matrix balancing. *IMA J. Numer. Anal*., 10.1093/imanum/drs019 (2013).

[58] Dunham I (2012). An integrated encyclopedia of DNA elements in the human genome. Nature.

[59] Plimpton S (1995). Fast parallel algorithms for short-range molecular dynamics. J. Comput. Phys..

[60] Kremer K, Grest GS (1990). Dynamics of entangled linear polymer melts: a molecular-dynamics simulation. J. Chem. Phys..

[61] Rosa, A. & Everaers, R. Structure and dynamics of interphase chromosomes. *PLoS Comput. Biol*., 10.1371/journal.pcbi.1000153 (2008). 10.1371/journal.pcbi.1000153 PMC2515109 18725929

[62] Allen, M. P. & Tildesley, D. J. Computer Simulation of Liquids (Oxford Science Publications) SE - Oxford science publications. *Oxford University Press* (1989).

