# Supplementary material for: Polymer physics indicates chromatin folding variability across single-cells results from state degeneracy in phase separation
Source: Nat Commun. 2020 Jul 3;11:3289. doi: 10.1038/s41467-020-17141-4 (PMC7335158; doi:10.1038/s41467-020-17141-4)
Supplement: Supplementary file 1 — Supplementary Information [file 41467_2020_17141_MOESM1_ESM.pdf]

Supplementary Materials

for

Polymer physics indicates chromatin folding variability across single-cells results from state degeneracy in phase-separation

Mattia Conte[1], Luca Fiorillo[1], Simona Bianco[1], Andrea M. Chiariello[1], Andrea Esposito[1], Mario Nicodemi[1,2,3,*]

[1]Dipartimento di Fisica, Università di Napoli *Federico II*, and INFN Napoli, Complesso Universitario di Monte Sant'Angelo, 80126 Naples, Italy.

[2]Berlin Institute for Medical Systems Biology, Max-Delbrück Centre (MDC) for Molecular Medicine, Berlin, Germany.

[3]Berlin Institute of Health (BIH), MDC-Berlin, Germany.

* Lead contact: mario.nicodemi@na.infn.it

SUPPLEMENTARY FIGURES

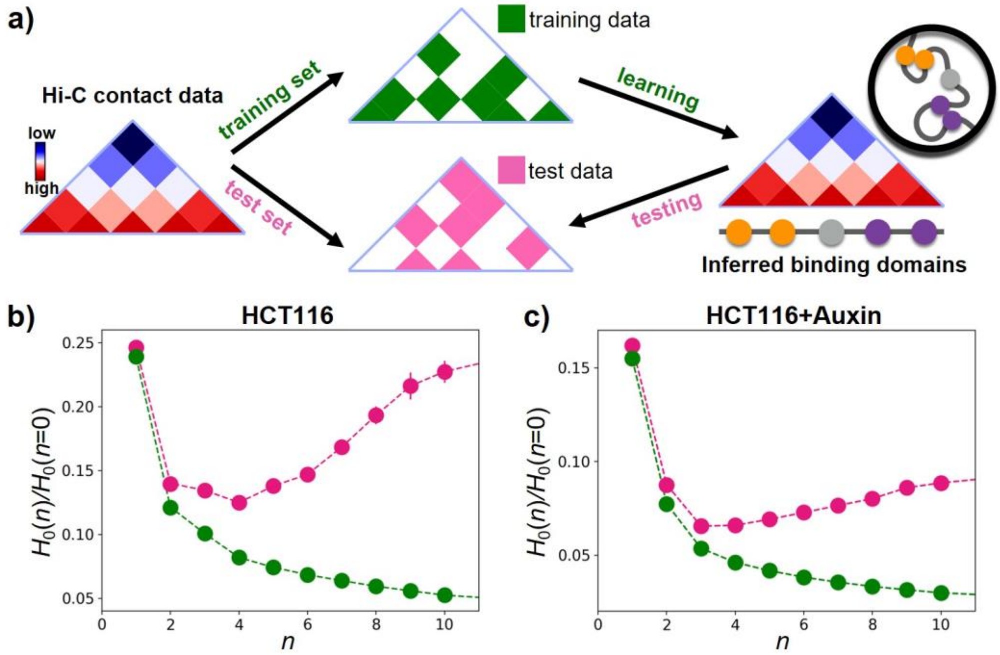

Supplementary Figure 1. The machine learning procedure used to infer the model binding sites.

a) Hi-C bulk contact data of a given locus are split in a training set (green) and a complementary test set (pink). Our machine learning procedure is run on the training dataset to infer the minimal SBS polymer model best describing the input bulk contact data. The test dataset is then used to test the model predicted contact frequencies. **b)** The cost function H_0, i.e., the normalized difference of model and experiment contact maps, is shown as a function of the number of different types of binding sites (colors), *n*, over the training (green) and the test (pink) sets of the model of HCT116 cells. Data are presented as mean values +/- s.e.m. by taking 20 independent simulations for each point. The value of *n* corresponding to the minimum of H_0 in the test set is where the model has its best predictive power. **c)** As in **b)**, for HCT116+Auxin cells.

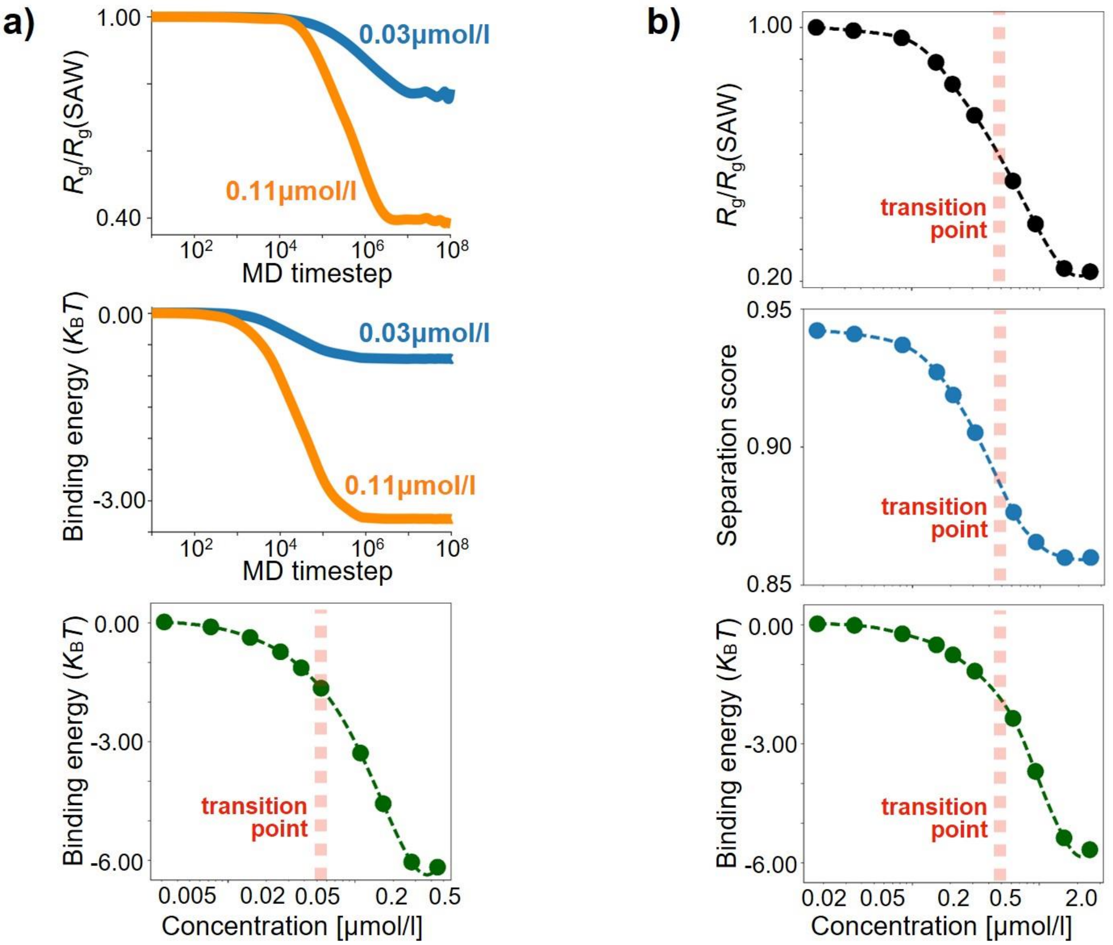

Supplementary Figure 2. The coil to globule phase separated state transition in the SBS model of the locus in HCT116 and HCT116+Auxin cells.

a) Top: The SBS model of the locus chr21:34.6Mb-37.1Mb in human HCT116 cells is thermalized into an equilibrium state from an initial SAW conformation at varying concentrations of the binders, as shown by the plateaued gyration radius and binding energy as a function of the MD time. In the case shown, the affinity of the specific binding sites is $3.1K_BT$, equal for all the different types, and the affinity of the un-specific binding sites is $2.7K_BT$. Bottom: The model has a phase transition from a coil to a globule phase separated state, marked by a sharp decrease of the gyration radius and separation score (see Fig. 1c) and of the binding energy of the system (shown here). **b)** The SBS model of the locus chr21:34.6Mb-37.1Mb in cohesin depleted HCT116 cells treated with Auxin (HCT116+Auxin) is thermalized to equilibrium at varying concentrations of the binders. The same affinities for specific and unspecific binding sites of the HCT116 model are used. The model undergoes a phase transition from a coil to a globule phase separated state, marked by a sharp decrease of its gyration radius (top), separation score (middle) and binding energy (bottom).

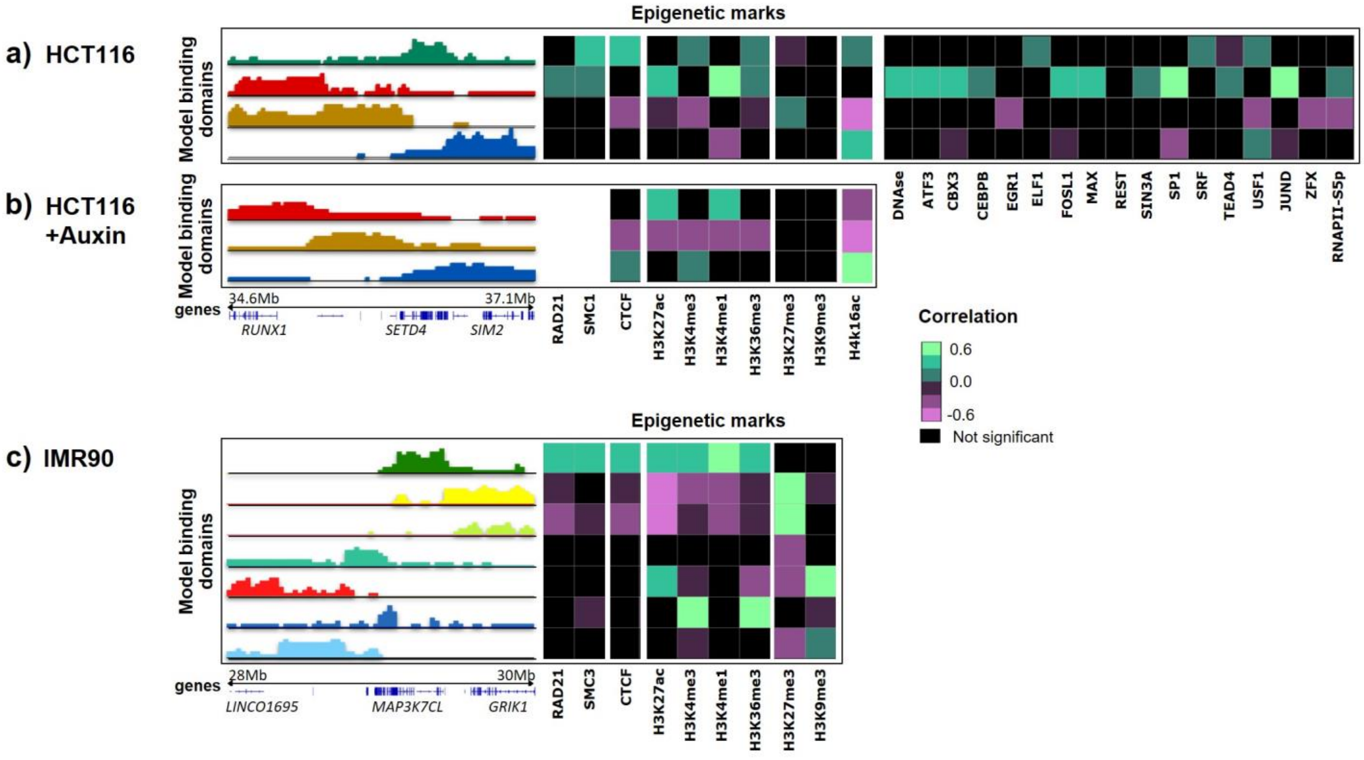

Supplementary Figure 3. Epigenetics signatures of the binding domains of the SBS model of the studied loci.

a) The binding domains of the SBS model of the locus chr21:34.6Mb-37.1Mb in human HCT116 cells (left) have statistically significant correlations each with a specific combinations of available chromatin architecture factors[1] (right). The rightmost panel includes additional factors from the ENCODE database[2] available in HCT116, but not in HCT116+Auxin cells. **b)** The binding domains of the SBS model of the locus chr21:34.6Mb-37.1Mb in human HCT116 cells treated with Auxin (HCT116+Auxin, left), corresponding to acute cohesin depletion, are shown with their significant correlations with epigenetics factors[1] (right). **c)** The binding domains of the SBS model of the locus chr21:28Mb-30Mb in human IMR90 cells (left) and their significant correlations with epigenetics factors[2] (right).

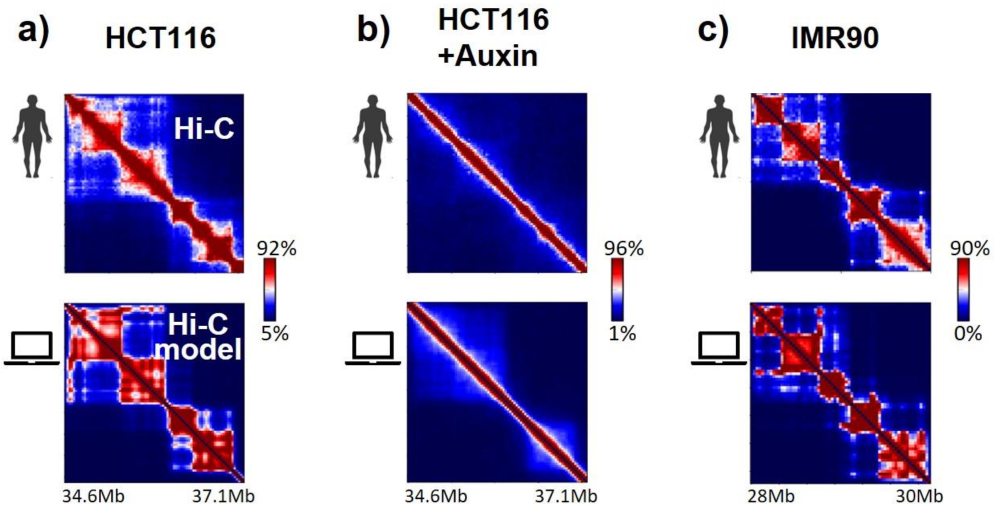

Supplementary Figure 4. Model and Hi-C average contact data have high correlations across cell types.

Hi-C (top) and model (bottom) average Hi-C contact maps in the studied loci have a high Pearson, r, and distance-corrected, r', correlations. In HCT116 cells (panel **a)**), we find r=0.88 and r'=0.68; in HCT116+Auxin cells (panel **b)**) r=0.93, r'=0.33; in IMR90 cells (panel **c)**) r=0.94, r'=0.74.

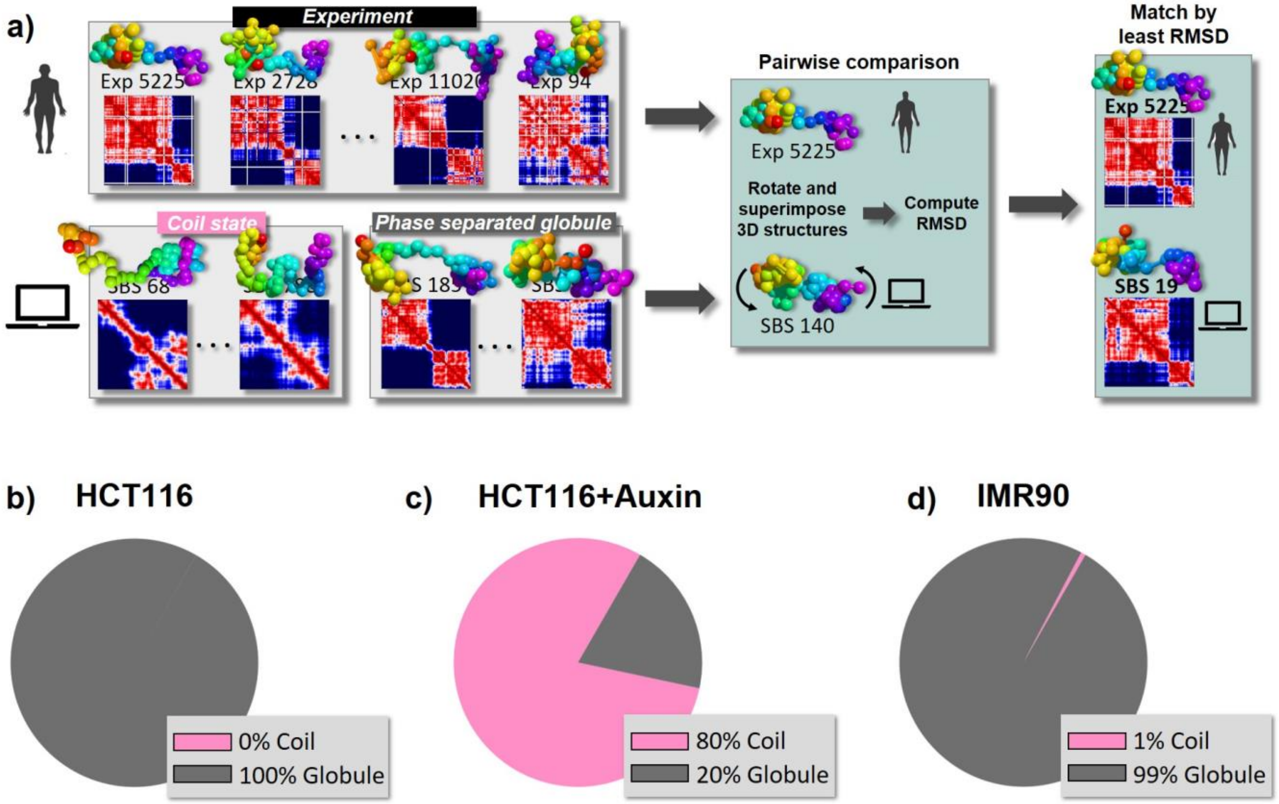

Supplementary Figure 5. Structural comparison of experimental and model 3D conformations by the least RMSD criterion.

a) Each 3D structure from imaging data[3] (top) is associated by the least RSMD criterion to a corresponding model 3D structure (bottom). **b)** In the HCT116 case, 100% of experimental imaged 3D structures is optimally mapped onto model structures in the globule phase separated state. **c)** In the HCT116+Auxin case, 20% of experimental imaged 3D structures is mapped onto model structures in the globule phase separated state and 80% onto the model coil conformations. **d)** In the IMR90 case, 99% of experimental imaged 3D structures is mapped onto model structures in the globule phase separated state.

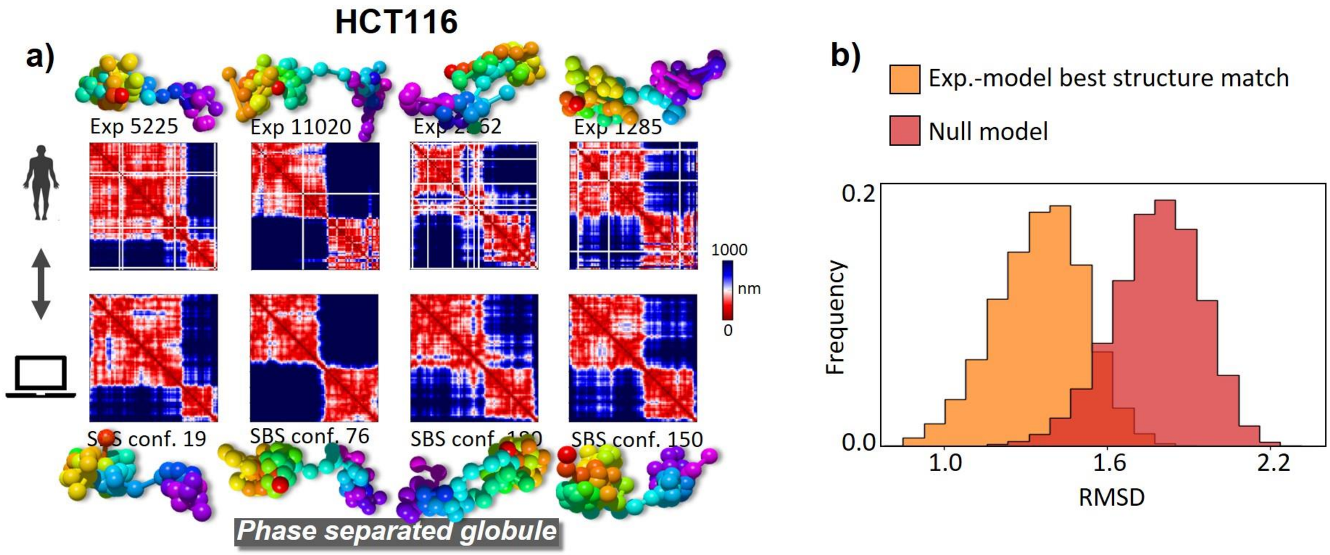

Supplementary Figure 6. Least RMSD best matches between experimental and model 3D structures in the case of the HCT116 locus.

a) The figure shows, for the HCT116 locus, examples of least RMSD best matches between 3D structures from imaging data[3] (top) and model 3D structures (bottom), along with the corresponding distance matrices. **b)** The association is statistically significant because the distribution of their RMDSs is different from the null model (p-value=0, two-sided Mann-Whitney test, see text). In the HCT116 case, we find that 100% of experimental structures map onto model conformations in the thermodynamics globule state (Supplementary Fig. 5b), consistent with our other analyses.

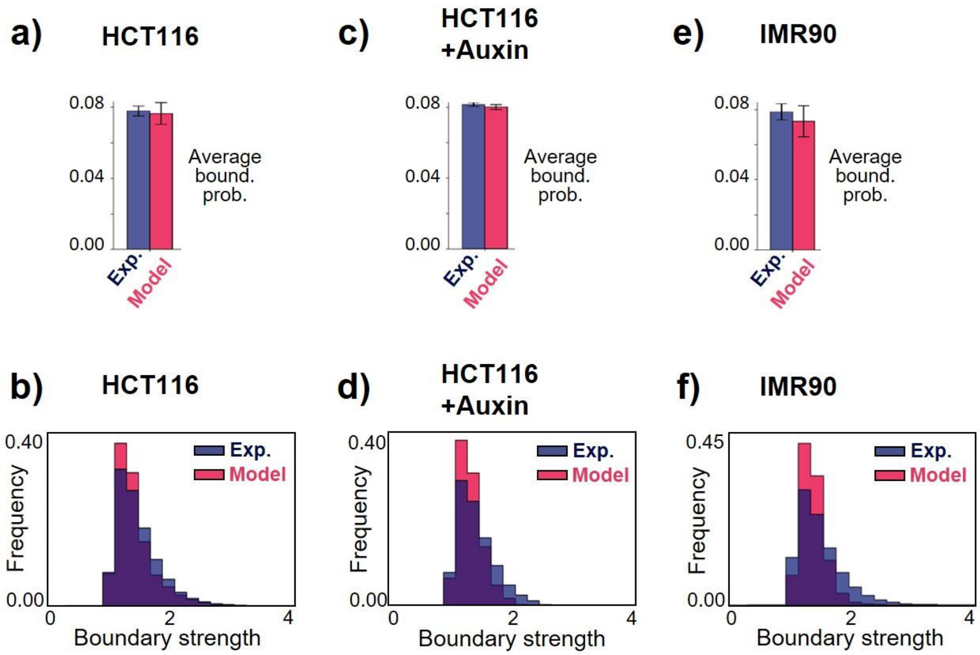

Supplementary Figure 7. Model and imaging single-molecule data have similar boundary probability and strength.

The average boundary probabilities (top, error bars s.e.m.) and the boundary strength distributions (bottom) are similar in super-resolution imaging experiments[3] and in the models of the studied loci in HCT116 (panel **a)**, **b)**), HCT116+Auxin (panel **c)**, **d)**) and IMR90 cells (panel **e)**, **f)**).

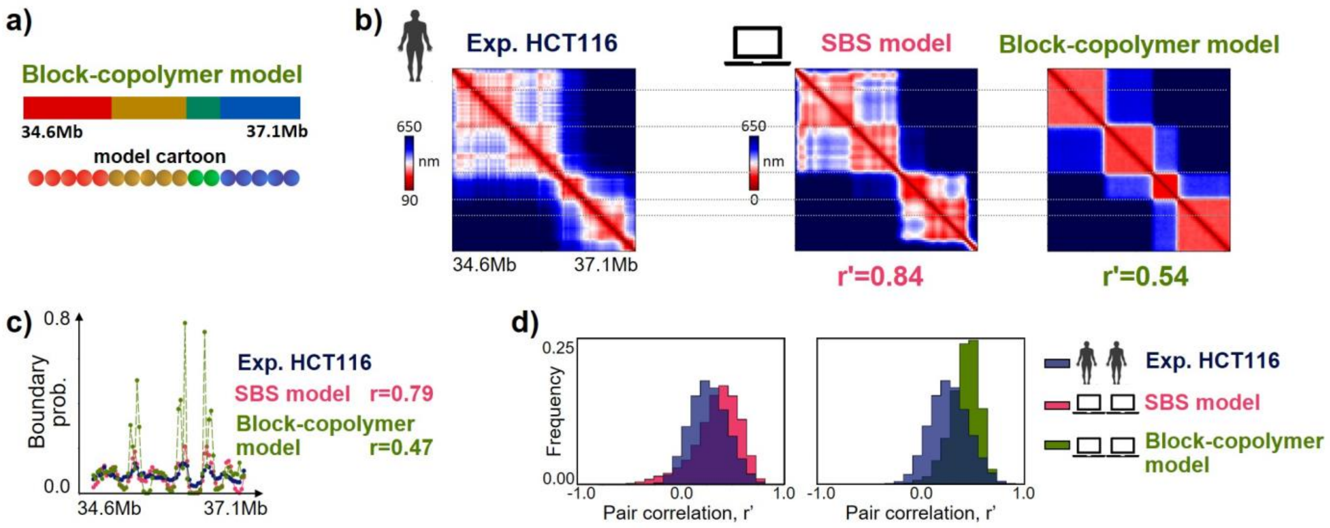

Supplementary Figure 8. The control block-copolymer model provides a poorer description of the complexity of experimental data than the SBS model.

a) A sketch of the linear arrangements of the different binding site types (colors) of the control block-copolymer model of the HCT116 locus. **b)** The contact pattern is shown (left) of the considered locus in HCT116 cells[3]. The SBS polymer model (center) matches well those data: their distance corrected Pearson correlation is r'=0.84. The control block-copolymer model (right) returns a poorer description of the complexity of experimental contact patterns (r'=0.54). **c)** The boundary probability of the control block-copolymer (green) also provides a poorer fit of real data (blue) than the SBS model (red). In particular, the control model peaks are four times higher than those from experiments and from the SBS model, showing that globule separation is much stronger in the control than in the SBS model. **d)** The pair-correlation between distance matrices from 3D structures of the SBS model (red) is closer to the experimental one (blue) than the block-copolymer model (green). The average value of r' in the experimental data (r'=0.3) is approximately equal to the SBS model value, whereas in the block-copolymer model it is 33% higher.

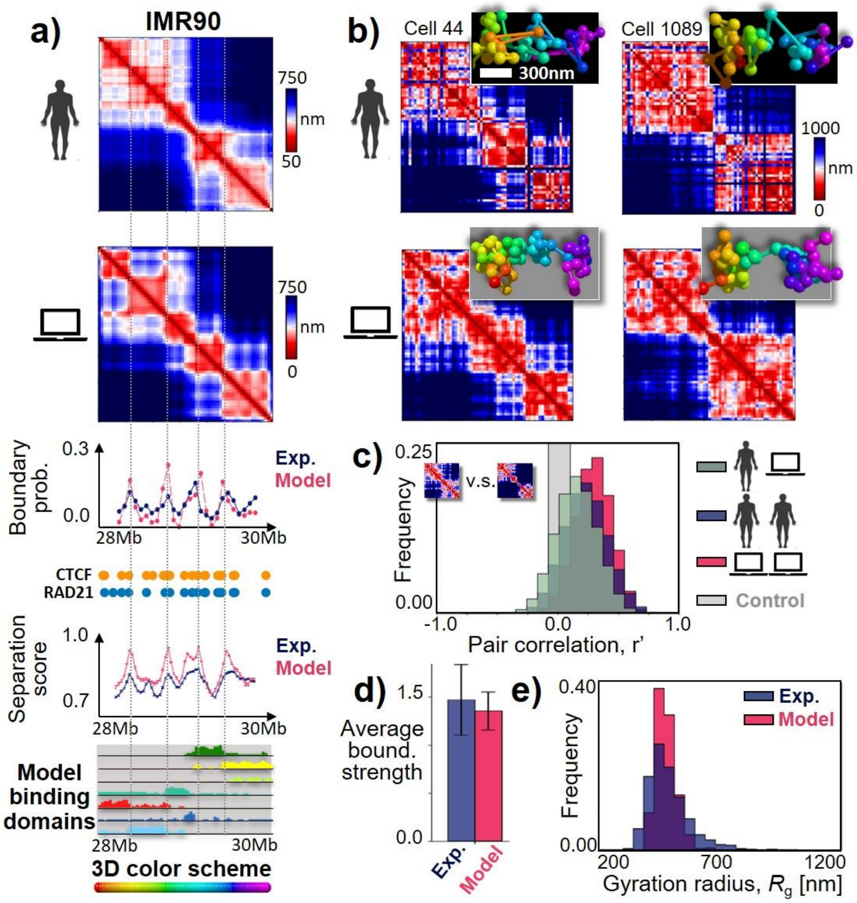

Supplementary Figure 9. Phase separation degeneracy explains variability of single-molecule conformations in IMR90 cells.

a) Top: In human IMR90 cells, the SBS model predicted median distance matrix of the 2Mb wide locus chr21:28Mb-30Mb in the globule separated state matches well independent super-resolution imaging data[3]; the Pearson and distance-corrected correlations are, respectively r=0.96 and r'=0.77. Middle: The probability of a domain boundary to be found in single-molecule 3D conformations along the locus (model-experiment correlation 0.60), the corresponding separation score (model-experiment correlation r=0.79) and the location of ChIP-seq CTCF (orange circles) and cohesin (RAD21, blue circles) sites[2]. Bottom: the intensity of the model specific binding site types along the locus, represented by different colors; the color scheme used in the 3D representations. **b)** Single-cell super-resolution 3D structures and their varying TAD-like domains (top) are statistically similar to model predicted single molecule conformations in the phase separated globule state (bottom, see text and Suppl. Figs. 5d, 10) whose variability results from the intrinsic thermodynamic state degeneracy. **c)** The distance-corrected correlation, r', between pairs of single-molecule distance matrices from imaging data has a broad distribution (blue) with an average of 0.23. It is statistically not distinguishable from the distribution of correlations between single-molecule imaged and model distance matrices (dark grey, two-sided Mann-Whitney p-value=0.02). The model-model correlation is in red and in grey a control. **d)** The average boundary strength is also similar in model and experiments (error bars s.d.). **e)** The model and experimental gyration radius distributions are also not distinguishable (p-value=0.68, two-sided Mann-Whitney test).

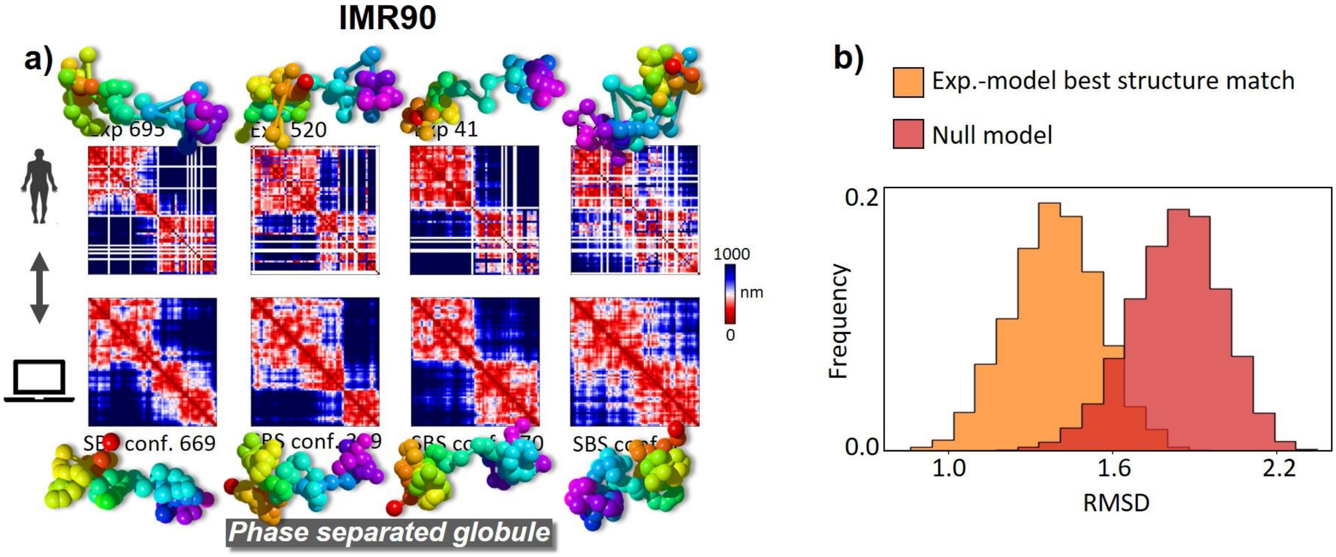

Supplementary Figure 10. Least RMSD best matches between experimental and model 3D structures in the case of the IMR90 locus.

a) The figure shows, for the IMR90 locus, examples of least RMSD best matches between 3D structures from imaging data[3] (top) and model 3D structures (bottom), along with the corresponding distance matrices. **b)** The association is statistically significant because the distribution of their RMDSs is different from the null model (p-value=0, two-sided Mann-Whitney test, see text). In the IMR90 case, we find that 99% of experimental structures map onto model conformations in the thermodynamics globule state, and 1% in the coil state (Supplementary Fig. 5d), consistent with our other analyses.

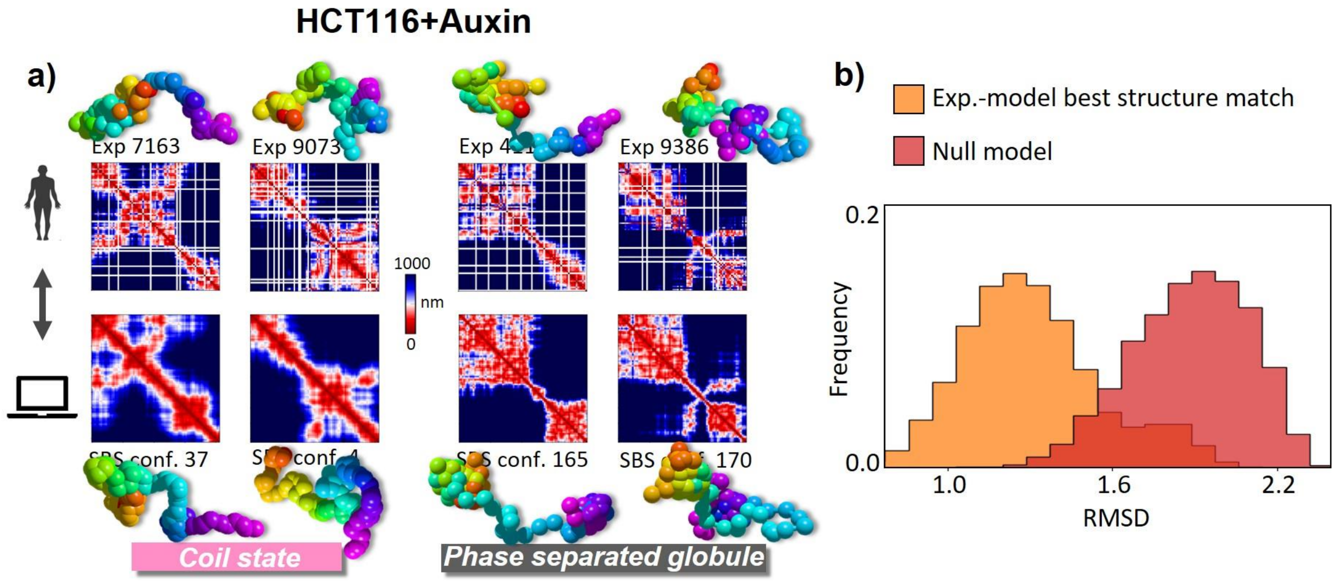

Supplementary Figure 11. Least RMSD best matches between experimental and model 3D structures in the case of the HCT116+Auxin locus.

a) The figure shows, for the HCT116+Auxin case, examples of least RMSD best matches between 3D structures from imaging data[3] (top) and model 3D structures (bottom), along with the corresponding distance matrices. **b)** The association is statistically significant because the distribution of their RMDSs is different from the null model (p-value=0, two-sided Mann-Whitney test, see text). In the HCT116+Auxin case, we find that 80% of experimental structures map onto model conformations in the thermodynamics coil state, and the remaining 20% in the globule state (Supplementary Fig. 5c), consistent with our other analyses.

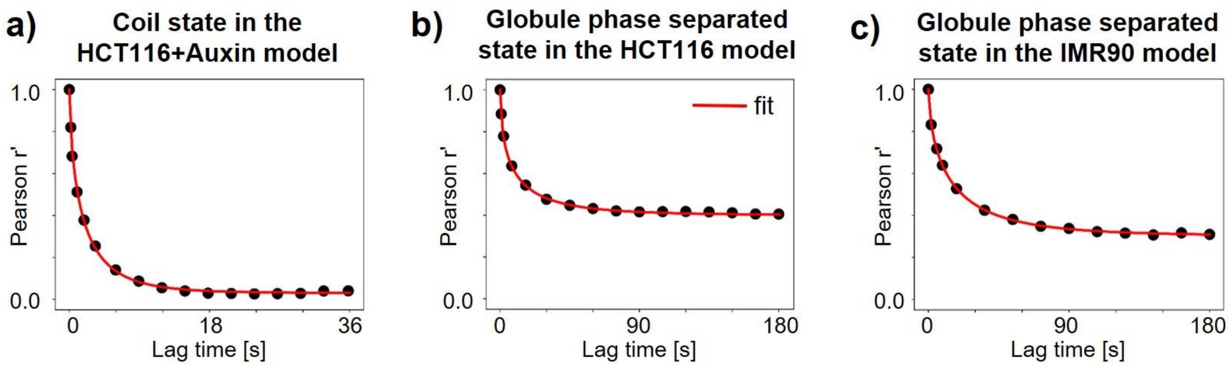

Supplementary Figure 12. Single-molecule distance correlation in time.

a) The time dependent correlation of the average distance-corrected Pearson correlation, r′, between single-molecule distance matrices is shown from MD simulations of the model of the HCT116+Auxin locus in the coil state. Superimposed is a stretched exponential fit. The long time plateau correlation level is 0.03 and the decay time is 9s. **b)** Same as in panel **a)**, for the model of the HCT116 locus in the globule phase separated state. The long time plateau correlation level is 0.39 and the decay time is 60s. **c)** Same as panel **a)** for the IMR90 locus model in the phase separated state. The long time plateau correlation level is 0.30 and the decay time is 90s.

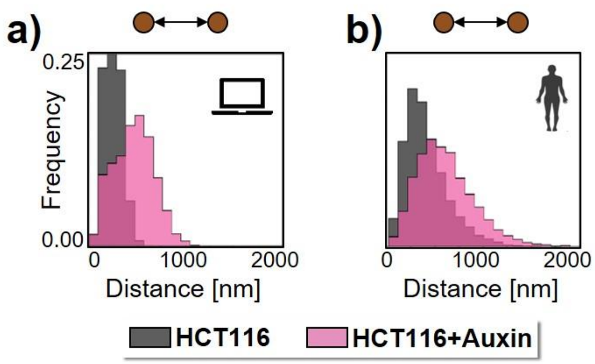

Supplementary Figure 13. Distance distributions in the HCT116 cell locus model and experiments.

a) The distance distribution of the brown pair of sites in the model of HCT116 and HCT116+Auxin cells. **b)** The experimental distance distributions of the brown pair[3] in HCT116 and in HCT116+Auxin cells.

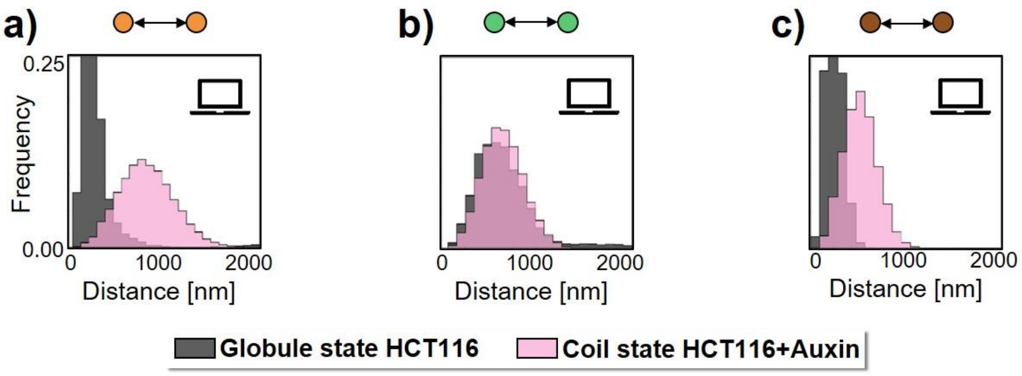

Supplementary Figure 14. Distance distributions of the pure states of the model of HCT116 and HCT116+Auxin cells.

The distance distribution of the orange (**a)**), green (**b)**) and brown (**c)**) pair of sites in the globule state of the HCT116 model and in the coil state of the HCT116+Auxin model.

SUPPLEMENTARY TABLES

HCT116	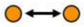	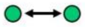
Exp.	330+-180	630+-280
Mod.	280+-200	620+-290

HCT116+Auxin	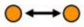	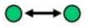
Exp.	760+-360	710+-340
Mod.	660+-260	620+-210

Supplementary Table I.

Average and standard deviation in nm of the physical distances of the site pairs investigated in Fig. 4 in the HCT116 and HCT116+Auxin models.

SUPPLEMENTARY REFERENCES

1. Rao, S. S. P. *et al.* Cohesin Loss Eliminates All Loop Domains. *Cell* **171**, 305-320.e24 (2017).

2. Dunham, I. *et al.* An integrated encyclopedia of DNA elements in the human genome. *Nature* **489**, 57–74 (2012).

3. Bintu, B. *et al.* Super-resolution chromatin tracing reveals domains and cooperative interactions in single cells. *Science (80-.).* **362**, eaau1783 (2018).
