# Supplementary material for: Polymer physics indicates chromatin folding variability across single-cells results from state degeneracy in phase separation
Source: Nat Commun. 2020 Jul 3;11:3289. doi: 10.1038/s41467-020-17141-4 (PMC7335158; doi:10.1038/s41467-020-17141-4)
Supplement: Supplementary file 2 — Reporting Summary [file 41467_2020_17141_MOESM2_ESM.pdf]

nature research

Corresponding author(s): Mario Nicodemi

Last updated by author(s): Jun 3, 2020

Reporting Summary

Nature Research wishes to improve the reproducibility of the work that we publish. This form provides structure for consistency and transparency in reporting. For further information on Nature Research policies, see Authors & Referees and the Editorial Policy Checklist .

Statistics

For all statistical analyses, confirm that the following items are present in the figure legend, table legend, main text, or Methods section.

n/a	Confirmed	
[ ]	[x]	The exact sample size (*n*) for each experimental group/condition, given as a discrete number and unit of measurement
[ ]	[x]	A statement on whether measurements were taken from distinct samples or whether the same sample was measured repeatedly
[ ]	[x]	The statistical test(s) used AND whether they are one- or two-sided<br>*Only common tests should be described solely by name; describe more complex techniques in the Methods section.*
[x]	[ ]	A description of all covariates tested
[x]	[ ]	A description of any assumptions or corrections, such as tests of normality and adjustment for multiple comparisons
[ ]	[x]	A full description of the statistical parameters including central tendency (e.g. means) or other basic estimates (e.g. regression coefficient) AND variation (e.g. standard deviation) or associated estimates of uncertainty (e.g. confidence intervals)
[ ]	[x]	For null hypothesis testing, the test statistic (e.g. *F*, *t*, *r*) with confidence intervals, effect sizes, degrees of freedom and *P* value noted<br>*Give P values as exact values whenever suitable.*
[x]	[ ]	For Bayesian analysis, information on the choice of priors and Markov chain Monte Carlo settings
[x]	[ ]	For hierarchical and complex designs, identification of the appropriate level for tests and full reporting of outcomes
[ ]	[x]	Estimates of effect sizes (e.g. Cohen's *d*, Pearson's *r*), indicating how they were calculated

Our web collection on statistics for biologists contains articles on many of the points above.

Software and code

Policy information about availability of computer code

Data collection	No software was used
Data analysis	We used the publicly available LAMMPS software for the Molecular Dynamics simulations of the SBS polymer modelling. Analyses involving the computation of spatial distances were performed using built-in functions within the Python SciPy software (version 1.3.1). The structural comparison of experimental and model conformations by RMSD was performed using the free available MDAnalysis Python library. We employed the POV-Ray software (version 3.7) to produce the 3D snapshots. Custom codes used to generate results reported in the manuscript can be made available from the corresponding upon request. All details of the algorithms are illustrated in the Methods section and in previous publications cited therein.

For manuscripts utilizing custom algorithms or software that are central to the research but not yet described in published literature, software must be made available to editors/reviewers. We strongly encourage code deposition in a community repository (e.g. GitHub). See the Nature Research guidelines for submitting code & software for further information.

Data

Policy information about availability of data

All manuscripts must include a data availability statement. This statement should provide the following information, where applicable:

- Accession codes, unique identifiers, or web links for publicly available datasets
- A list of figures that have associated raw data
- A description of any restrictions on data availability

The data supporting the findings of this study are available from the corresponding author upon request.

Field-specific reporting

Please select the one below that is the best fit for your research. If you are not sure, read the appropriate sections before making your selection.

☒ Life sciences ☐ Behavioural & social sciences ☐ Ecological, evolutionary & environmental sciences

For a reference copy of the document with all sections, see nature.com/documents/nr-reporting-summary-flat.pdf

Life sciences study design

All studies must disclose on these points even when the disclosure is negative.

Sample size	Our study is based on polymer physics computer simulations. Our analyses were performed using for each studied locus a statistical ensemble of 1000 independent polymer conformations, as described in detail in the Methods section of the manuscript.
Data exclusions	No data were excluded.
Replication	Our investigation is based on computer simulations and we do not generate new biological data. We verified the reproducibility of the finding of our manuscript running an additional battery of 200 independent numerical simulations for each studied locus.
Randomization	Randomization is not relevant to our study, since our investigation is based on computer simulations.
Blinding	Blinding is not applicable to our study, since our investigation is based on computer simulations and we do not generate new biological data.

Reporting for specific materials, systems and methods

We require information from authors about some types of materials, experimental systems and methods used in many studies. Here, indicate whether each material, system or method listed is relevant to your study. If you are not sure if a list item applies to your research, read the appropriate section before selecting a response.

Materials & experimental systems

n/a	Involved in the study
☒	☐ Antibodies
☒	☐ Eukaryotic cell lines
☒	☐ Palaeontology
☒	☐ Animals and other organisms
☒	☐ Human research participants
☒	☐ Clinical data

Methods

n/a	Involved in the study
☒	☐ ChIP-seq
☒	☐ Flow cytometry
☒	☐ MRI-based neuroimaging
